# Diagnostic accuracy of machine learning for endometriosis: a systematic review and meta-analysis

**DOI:** 10.3389/fendo.2025.1735567

**Published:** 2026-01-27

**Authors:** Bingyi Zhang, Xiaoli Lv, Dan Li, Longtao Zhang, Ziyang Ru, Yuxia Ma

**Affiliations:** School of Acupuncture and Tuina, Shandong University of Traditional Chinese Medicine, Jinan, Shandong, China

**Keywords:** diagnosis, endometriosis, machine learning, meta-analysis, systematic review

## Abstract

**Background:**

Researchers have explored machine learning (ML) in diagnosing endometriosis. However, systematic evidence on its diagnostic accuracy for endometriosis remains scarce.

**Objective:**

To systematically review the performance of machine learning for the diagnosis of endometriosis.

**Search strategy:**

PubMed, Embase, Cochrane Library, and Web of Science were systematically searched up to October 11, 2024.

**Selection criteria:**

Studies that constructed machine learning models to diagnose endometriosis.

**Data collection and analysis:**

Two reviewers independently screened studies, extracted data, and assessed study quality. The risk of bias of the included studies was assessed using the Prediction Model Bias Risk Assessment Tool.

**Main results:**

A total of 45 publications were included. Participant numbers ranged from 39 to 612,777. A meta-analysis showed that the area under the curve (AUC), sensitivity, and specificity of models based on clinical features were 0.810 (95% confidence interval [CI]: 0.786–0.835), 0.81 (95% CI: 0.77–0.84), and 0.76 (95% CI: 0.73–0.79) in the training sets, and 0.796 (95% CI: 0.770–0.822), 0.80 (95% CI: 0.75–0.84), and 0.76 (95% CI: 0.72–0.80) in the validation sets. The AUC, sensitivity, and specificity of models based on genetic information were 0.982 (95% CI: 0.975–0.990), 0.94 (95% CI: 0.90–0.97), and 0.99 (95% CI: 0.94–1.00) in the training sets. For the validation sets, these metrics were 0.865 (95% CI: 0.701–1.000), 0.83, and 0.59–0.96. Models based on imaging features exhibited an AUC of 0.979 (95% CI: 0.959–0.999) and 0.983 (0.971–0.995) in the training and validation sets, respectively.

**Conclusions:**

ML models, particularly those based on genetic information and imaging, possess substantial accuracy for detecting endometriosis.

**Systematic Review Registration:**

https://www.crd.york.ac.uk/prospero/, identifier CRD42024605113.

## 1 Introduction

Endometriosis, a chronic inflammatory gynecological condition, is characterized by the ectopic growth of endometrial-like tissue outside the uterus, such as in pelvic organs (1). Its global prevalence is notable, affecting 8%-10% of women of reproductive age and up to 50% of those experiencing infertility (2). Associated severe clinical manifestations, including infertility and pain, significantly impact individuals’ daily lives and mental well-being (3). Furthermore, Taylor HS et al.’s research shows that this condition necessitates lifelong management and frequently presents with comorbidities, leading to substantial healthcare resource utilization (4). Prolonged diagnostic delays contribute significantly to this burden (5). Consequently, effective early diagnosis and preventative measures are urgently needed to enhance diagnostic efficiency.

Endometrial-like tissue can appear in multiple body sites, including extra-pelvic regions, though it primarily localizes within the pelvis (6). Pathologically, while various hypotheses exist regarding the origin and pathogenesis of ectopic lesions, such as retrograde menstruation and coelomic metaplasia, the definitive etiology remains incompletely elucidated (4). Current diagnostic gold standards rely on laparoscopic visualization of lesions combined with histopathological confirmation. However, this invasive procedure, due to its invasiveness and surgical risks, is unsuitable for early screening (5). Moreover, the long-term concealment of symptoms often results from pain rationalization attributed to personal and sociocultural factors (7). For individuals with mild endometriosis, small lesions may be difficult to identify laparoscopically. The benefit-to-risk ratio of surgery also warrants careful consideration. These factors collectively contribute to diagnostic delays (median: 5–12 years) and increased misdiagnosis rates, significantly exacerbating the disease burden (8). Researchers are therefore increasingly focusing on noninvasive diagnostic markers for early detection, encompassing demographic features, biomarkers, omics data, and imaging modalities. Despite numerous investigations, single-factor diagnostic accuracy has not yet reached clinically practical levels. Notably, combined diagnostic models incorporating multiple indicators appear to be a promising strategy (9).

Machine learning (ML), a technology with substantial clinical translational potential in artificial intelligence (AI), has demonstrated unique advantages in integrating healthcare big data by constructing nonlinear feature association models (10). It offers novel avenues for early endometriosis diagnosis by establishing noninvasive diagnostic systems that integrate ultrasound imaging, serum protein markers, and patient phenotype data. While some researchers have explored the diagnostic accuracy of ML in endometriosis, systematic evidence demonstrating its efficacy is lacking. Thus, this investigation was conducted to systematically evaluate the clinical utility of existing endometriosis diagnostic models using a quantitative meta-analysis. Furthermore, the present study aims to identify key diagnostic potential factors, thereby providing evidence for AI-assisted diagnosis.

## 2 Methods

### 2.1 Study registration

This investigation adhered to the Preferred Reporting Items for Systematic Reviews and Meta-Analyses (PRISMA) guidelines (11). The study protocol received approval following registration with the International Prospective Register of Systematic Reviews (PROSPERO) (CRD42024605113).

### 2.2 Eligibility criteria

Inclusion criteria:

- Cross-sectional, cohort, and case-control study designs.
- Research constructing machine learning models (MLMs) for diagnosing endometriosis.
- Literature published in English.

Exclusion criteria:

- Study types such as meta-analyses, reviews, guidelines, expert opinions, and conference abstracts that are not full-text publications.
- Investigations focusing solely on risk factor analysis without developing a complete MLM.
- ML accuracy was evaluated using any metrics listed below: AUC, sensitivity, specificity, accuracy, precision, confusion matrix, F1-score, and calibration curve. Studies developing MLMs should include at least one of these metrics to evaluate model performance; otherwise, the study was excluded.
- Studies assessing single-factor prediction accuracy.

### 2.3 Data sources and search strategy

We conducted a systematic retrieval of the Cochrane Library, Web of Science, Embase, and PubMed up to October 11, 2024. We adopted a subject term plus free term search method, and we did not restrict the search by region or period. We employed the keywords ‘Endometriosis’ and ‘Machine learning’ as well as their synonyms. All eligible articles underwent peer review. A comprehensive search strategy for all databases is detailed in **Supplementary Table S1**.

### 2.4 Study selection and data extraction

We imported the retrieved literature into EndNote and read the titles and abstracts after eliminating duplicates. Then, we screened the original studies that met the criteria for our systematic review and downloaded the full texts. Finally, we reviewed the full texts and selected the eligible studies. Before data extraction, we created a standard spreadsheet that included the following: title, first author, publication year, author’s country, research type, patient source, diagnostic criteria for endometriosis, number of endometriosis cases, total number of cases, number of endometriosis cases in the training set, total number of cases in the training set, generation method of validation set, overfitting method, number of endometriosis cases in the validation set, number of cases in the validation set, missing value processing method, variable screening/feature selection method, type of model used, modeling variables, AUC, area under the receiver operating characteristic curve (AUC), diagnostic 2x2 tables, sensitivity, specificity, and precision.

Two researchers carried out the above literature screening and data extraction independently and then cross-checked. If there was any dispute, the third researcher helped to decide.

### 2.5 Risk of bias in studies

The risk of bias (ROB) in eligible studies was appraised using the Prediction Model Risk of Bias Assessment Tool (PROBAST) (12). PROBAST is a framework that assesses overall ROB and applicability across four domains: participant selection, predictor variables, outcome assessment, and statistical analysis. Each domain contains specific questions that are evaluated as having a low, high, or unclear ROB. A domain is considered to be at high risk if any of its questions are at high risk. A domain is considered to be of low risk if all of its questions are of low risk. If a domain lacks high risk but includes unclear risks, it is categorized as having an unclear risk.

Two researchers evaluated the ROB independently based on PROBAST, and then cross-checked their findings. Any disagreements were resolved with the help of a third researcher.

### 2.6 Synthesis methods

A meta-analysis of the AUC, a measure of overall MLM accuracy, was performed. For studies lacking the AUC or its 95% confidence interval (CI) and standard error, their standard error and 95% CI were estimated following Debray TP et al. (13). Heterogeneity among studies was assessed using the I² statistic. A random-effects model was employed for meta-analysis when I² > 50%, while a fixed-effects model was used when I² < 50%.

Sensitivity and specificity were also meta-analyzed using a bivariate mixed-effects model. Diagnostic 2x2 tables are essential for meta-analysis. When these tables were unreported in original studies, calculations were derived from case numbers combined with sensitivity, specificity, and precision. Subgroup analyses were conducted based on dataset, model type, and modeling variable types. Meta-analyses were executed using Stata software. A P-value below 0.05 indicated statistical significance.

## 3 Results

### 3.1 Study selection

**Figure 1:**
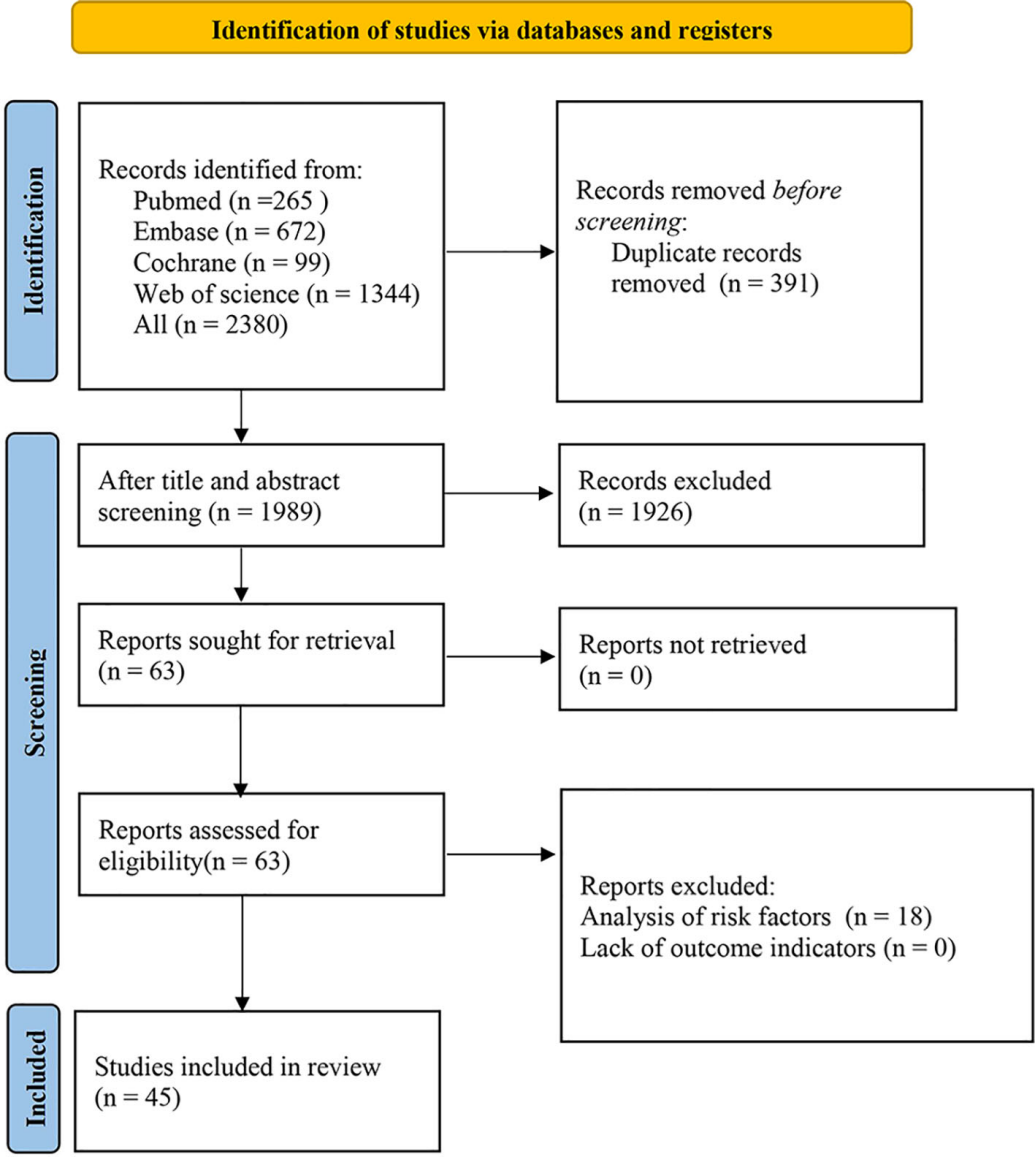
PRISMA flow diagram for literature screening.

The database retrieval yielded 2,380 articles. After removing 391 duplicates based on titles, 1,989 articles were screened by title and abstract. Of those, 1,926 were excluded for being irrelevant, reviews, letters, case reports, experiments, registered protocols, non-English publications, or other reasons. The remaining 63 articles were downloaded for a full-text review. Eighteen articles were excluded for risk factor analysis or missing outcome metrics. Finally, 45 articles passed the full-text evaluation. **Figure 1** illustrates the study selection process according to PRISMA guidelines.

### 3.2 Study characteristics

The 45 eligible articles were published between 2003 and 2024, with 28 (62%) published in the last five years. Fourteen investigations were conducted in China, while others originated from 17 countries, including France, the United Kingdom, Poland, Germany, Russia, the United States, Spain, Israel, Italy, the Netherlands, Canada, Kazakhstan, Austria, Turkey, Belgium, and Portugal. All studies employed a case-control design. The age of women was within the reproductive range. Eight studies (17.8%) focused on individuals with infertility (14–21). Thirty-one studies incorporated women with relevant symptoms but unclear endometriosis diagnosis as controls. Conversely, two studies utilized completely healthy women as controls (22, 23). Participant numbers ranged from 39 to 612,777 (17, 24). Seventeen studies (38%) underwent internal validation, such as random sampling or k-fold cross-validation. Only six studies (13%) performed external validation. A total of 79 models demonstrating the most effective outcome prediction were identified. Sixty-nine models (87%) reported the AUC, and 74 models (94%) reported diagnostic 2x2 tables or sensitivity and specificity metrics. Twenty-one ML methods were adopted to construct diagnostic models. Logistic regression (LR) was the most frequent (39%), followed by random forest (RF) (14%). Feature selection identified the most relevant features for effective and interpretable models. The current research explored target features for endometriosis diagnosis, including clinical characteristics, genetic regulators, imaging, and omics data. Clinical features, such as questionnaires, medical records, and serum/urine biomarkers, constituted the majority of features in all studies. Genetic regulator information primarily came from salivary microRNAs (miRNAs), with Sofiane Bendifallah and colleagues contributing multiple sequential studies (25–28). Other feature types were less prevalent (**Supplementary Tables S2**–**S4**).

### 3.3 Risk of bias in studies

**Figure 2:**
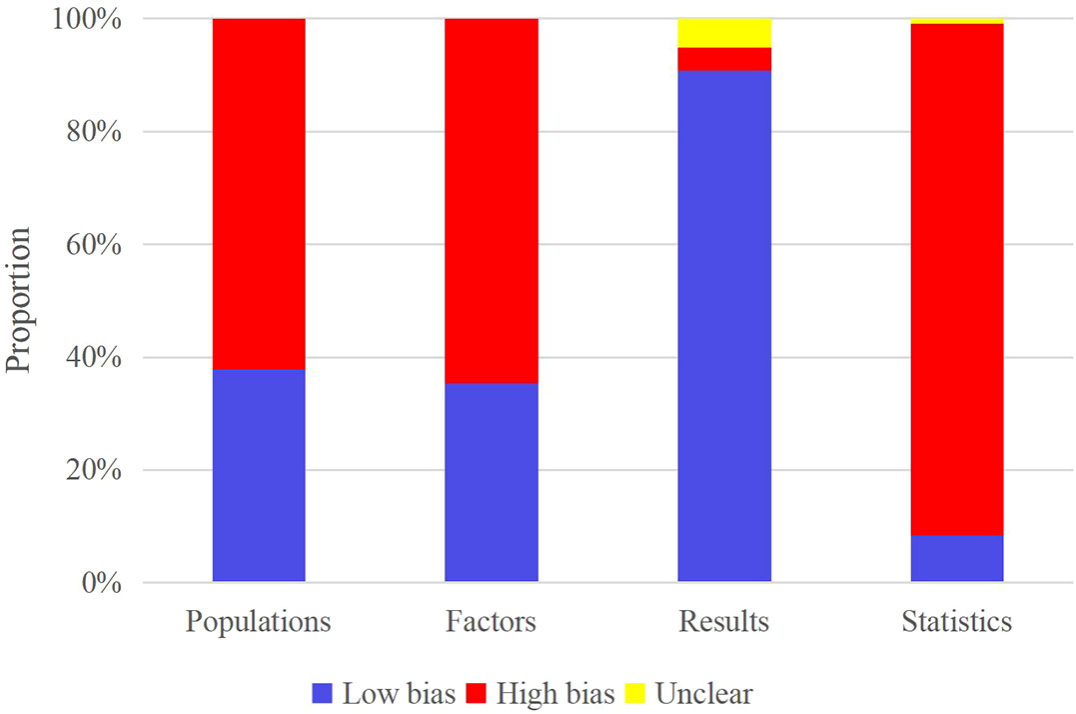
ROB evaluation results for included models using PROBAST.

PROBAST was utilized to appraise the ROB of predictive diagnostic models. Of the 45 eligible studies, most were case-control or prospective cohort studies from registry databases. Among 118 models, 44 exhibited low ROB concerning study subjects, 42 regarding predictor variables, 107 regarding outcomes, and 10 regarding statistical analysis (**Figure 2**). Overall, certain models presented a high ROB, particularly in statistical analysis. This suggests that future research should prioritize optimization and validation of statistical analysis methods.

### 3.4 Meta-analysis

#### 3.4.1 ML based on clinical features

To elucidate the heterogeneous impact of datasets, feature types, and ML methods on constructing endometriosis diagnostic models, a subgroup analysis was performed on 118 extracted models. Among models reporting AUC, 55 were in the training set and 31 were in the internal or external validation sets. LR models constituted the largest proportion in both the training and validation sets, at 22 (40%) and 17 (54.8%), respectively. The pooled AUC for all training set models was 0.810 (95% CI: 0.786-0.835), while for the validation sets, it was 0.796 (95% CI: 0.770-0.822). In the validation set, the top five ML methods, ranked from highest to lowest, were VoteClassifier (AUC = 0.911, n = 2), RF (AUC = 0.831, n = 2), least squares-support vector machine (SVM) (AUC = 0.803, n = 3), extreme gradient boosting (XGBoost) (AUC = 0.802, n = 3), and LR (AUC = 0.788, n = 17) (**Table 1**, **Supplementary Figures S1**, **S2**).

**Table 1:** Meta-analysis results of AUC for ML-based diagnosis of endometrial heterogeneity in training and validation sets.

Moldes	Training set						Validation set					
	n	Events	Sample size	AUC (95% CI)	I2	tau2	n	Events	Sample size	AUC (95% CI)	I2	tau2
Clinical												
LR	22	11784	506167	0.820(0.773 - 0.868)	98.4	0.0132	17	2678	4942	0.788(0.761 - 0.815)	75.2	0.0022
DT	4	5316	492177	0.768(0.682 - 0.855)	98.5	0.0070	1	87	100	0.780(0.714 - 0.846)	NA	NA
LightGBM	3	1556	12471	0.810(0.752 - 0.868)	78.6	0.0020	2	1316	11981	0.711(0.489 - 0.932)	96.7	0.0247
NB	1	74	222	0.810(0.753 - 0.867)	NA	NA	–					
RF	7	5915	505149	0.810(0.722 - 0.897)	99.0	0.0130	2	153	212	0.831(0.646 - 1.000)	92.0	0.0164
ANN	2	337	668	0.773(0.688 - 0.857)	81.2	0.0030	1	66	112	0.744(0.650 - 0.838)	NA	NA
SVM	5	848	1432	0.802(0.752 - 0.852)	84.1	0.0030	3	220	348	0.803(0.747 - 0.859)	48.5	0.0012
XGBoost	3	1835	492401	0.825(0.695 - 0.955)	99.6	0.0130	3	1182	122778	0.802(0.648 - 0.956)	97.9	0.0177
AdaBoost	2	4590	491107	0.828(0.612 - 1.000)	99.9	0.0244	–			-		
LASSO	1	157	313	0.850(0.813 - 0.887)	NA	NA	–			-		
VoteClassifier	2	2252	3468	0.815(0.688 - 0.943)	99.2	0.0080	2	174	200	0.911(0.880 - 0.942)	0.0	<0.0001
NB	1	74	222	0.810(0.753 - 0.867)			–					
KNN	2	141	422	0.777(0.733 - 0.821)	0.0	0.0000	–			-		
Overall	55	34879	2506219	0.810(0.786 - 0.835)	98.7	0.0080	31	5876	140673	0.796(0.770 - 0.822)	90.8	0.0044
Genetic												
LR	1	153	200	0.968(0.949 - 0.987)	NA	NA						
RF	3	328	553	0.984(0.968 - 1.000)	54.5	0.0001	1	24	48	0.939(0.877 - 1.000)		
XGBoost	1	153	200	0.984(0.971 - 0.997)	NA	NA						
AdaBoost	1	153	200	0.984(0.971 - 0.997)	NA	NA						
NNET							1	18	36	0.770(0.636 - 0.904)		
Overall	6	787	1153	0.982(0.975 - 0.990)	29.9	<0.0001	2	42	84	0.865(0.701 - 1.000)	80.1	0.0114
Imaging												
LightGBM	2	320	652	0.991(0.984 - 0.997)	0.0	<0.0001	2	80	162	0.980(0.962 - 0.999)	33.1	0.0001
DL	1	576	790	0.900(0.850 - 0.950)	NA	NA	2	31	45	0.986(0.963 - 1.000)	0.0	<0.0001
Overall	3	448	1442	0.979(0.959 - 0.999)	85.0	0.0002	4	111	207	0.983(0.971 - 0.995)	0.0	<0.0001
Omics												
LR	1	23	39	0.870(0.776 - 0.964)	NA	NA						
RF	1	21	41	0.886(0.783 - 0.989)	NA	NA						
SVM							1	20	26	0.833(0.712 - 0.954)	0.0	<0.0001
Overall	2	44	80	0.877(0.808 - 0.947)	0.0	<0.0001	1	20	26	0.833(0.712 - 0.954)	0.0	<0.0001

Of the models reporting sensitivity and specificity, 56 originated from the training sets and 30 from the validation sets. LR remained the most common method, accounting for 24 models (43%) in the training sets and 16 models (53%) in the validation sets. The pooled sensitivity for the training set models was 0.81 (95% CI: 0.77-0.84), and the specificity was 0.76 (95% CI: 0.73-0.79) (**Supplementary Figures S3**–**S5**). The pooled sensitivity for validation set models was 0.80 (95% CI: 0.75-0.84), and the specificity was 0.76 (95% CI: 0.72-0.80) (**Supplementary Figure S6**). Diagnostic performance across all dataset groups was good, with sensitivity ranging from 0.49 to 0.98 and specificity ranging from 0.59 to 1.00. In the validation set, the common ML method LR achieved a sensitivity of 0.78 and specificity of 0.75 (**Table 2**).

**Table 2:** Meta-analysis results of sensitivity and specificity for ML in diagnosing endometrial heterogeneity in training and validation sets.

Moldes	Training set			Validation set		
	n	Sen (95% CI)	Spe (95% CI)	n	Sen (95% CI)	Spe (95% CI)
Clinical						
LR	24	0.77(0.74,0.81)	0.79(0.74,0.83)	16	0.78(0.72,0.84)	0.75(0.69,0.79)
DT	5	0.78(0.67,0.85)	0.76(0.69,0.81)	1	0.91	0.66
LightGBM	3	0.73-0.97	0.73-0.97	3	0.49-0.88	0.76-1
NB	1	0.72	0.77			
RF	7	0.83(0.68,0.92)	0.72(0.69,0.75)	2	0.67-0.92	0.74-0.92
ANN	2	0.62-0.72	0.73-0.75	1	0.66	0.78
SVM	2	0.85(0.76,0.90)	0.70(0.59,0.78)	2	0.70-0.82	0.69-0.75
XGBoost	3	0.85(0.76-0.90)	0.70(0.59-0.78)	3	0.69-0.93	0.63-0.92
AdaBoost	2	0.64-0.93	0.66-0.95			
VoteClassifier hard	2	0.95-0.98	0.6-0.8	2	0.91-0.93	0.88-0.92
KNN	2	0.66-0.86	0.67-0.71			
Overall	56	0.81(0.77,0.84)	0.76(0.73,0.79)	30	0.80(0.75,0.84)	0.76(0.72,0.80)
Genetic						
LR	1	0.94	1			
RF	4	0.96(0.90,0.98)	0.98(0.95,0.99)	1	0.83	0.96
XGBoost	1	0.97	1			
Adaboost	1	0.97	1			
ANN	1	0.72	0.72	1	0.83	0.59
Overall	8	0.94(0.90,0.97)	0.99(0.94,1.00)	2	0.83	0.59-0.96
Imaging						
LightGBM	2	0.96	0.95-0.97	2	0.88	0.97-1
DL	1	0.9	0.9	2	1.00	0.9-1
Overall	3	0.96	0.90-0.97	4	0.89(0.82,0.94)	0.99(0.93,1.00)
Omics						
LR	1	0.81	0.85			
DT	2	0.78-0.9	0.59-0.81			
ANN				1	0.92	0.90
GA	1	0.97	0.94			
RF	1	0.85	0.8			
QC	1	0.73	0.77			
SVM				1	0.81	1.00
Overall	6	0.86(0.77,0.92)	0.81(0.71,0.88)	2	0.81-0.92	0.90-1.00

#### 3.4.2 ML based on genetic regulators

Among the genetic regulator group, six training set models reported AUC, yielding a pooled AUC of 0.982 (95% CI: 0.975-0.990). RF was the most frequently used model (n = 3, AUC = 0.984). The other models were XGBoost (AUC = 0.984), adaptive boosting (AdaBoost) (AUC = 0.984), and LR (AUC = 0.968). The validation set included two models, RF (AUC = 0.939) and neural network (NNET) (AUC = 0.770). The pooled AUC was 0.865 (95% CI: 0.701-1.000). Eight of the models reporting sensitivity and specificity were from training sets. The pooled sensitivity was 0.94 (95% CI: 0.90-0.97), and the specificity was 0.99 (95% CI: 0.94-1.00). RF was the predominant ML method (n = 4, sensitivity = 0.96, specificity = 0.98). No ML method constructed more than one model in the validation set. Sensitivity across all datasets ranged from 0.72 to 0.97, and specificity ranged from 0.59 to 1.00 (**Tables 1**, **2**).

#### 3.4.3 ML based on radiomics

Three studies utilized ultrasound images as input to create diagnostic models for subjects with ovarian endometriotic cysts (OEC) against various controls. Specific control groups comprised subjects with benign mucinous cystadenoma, ovarian teratoma, and tubo-ovarian abscess. Kuo Miao et al. and Ping Hu et al. employed data-augmentation-based deep learning (DL) methods to differentiate OEC from specific ovarian lesions. Kuo Miao et al. (32) introduced a DL architecture that achieved an AUC of 0.90 on 1,153 images. Ping Hu et al. (33) compared various convolutional neural network (CNN) models for identifying tubo-ovarian abscess and OEC, including ResNet-152, DenseNet-161, and EfficientNet-B7. ResNet-152 achieved an AUC of 0.986 on an independent test set, significantly outperforming physician diagnoses (AUC = 0.683-0.781) and the CA125 marker (AUC = 0.564). Lu Liu et al. (34) filtered 22 radiomic features using the Least Absolute Shrinkage and Selection Operator (LASSO) regression to construct a classification system with LightGBM and LR to differentiate OEC from ovarian teratoma. In their study, the LR model demonstrated superior performance on the test set (AUC = 0.981), while LightGBM exhibited the highest specificity (0.971).

#### 3.4.4 ML based on other omics

Due to the limited number of studies on other omics, quantitative analysis was not applicable. Among the included studies, six studies (17, 21, 35–38) employing ML techniques for the diagnosis of endometriosis were trained on proteomics, lipomics, or microbiomics features. Three studies (21, 35, 37) focused on proteomics to determine if women with symptoms had endometriosis. Monika (35) constructed a diagnostic model using a decision tree algorithm to automatically identify a specific pattern of mass peaks with a sensitivity of 78.4% and a specificity of 59.0%. L. Wang (37) developed three ML methods: GA, DTA, and QC, which were trained using urine specimens. They reported that the GA model was superior to the latter two methods, achieving the highest sensitivity (96.7%) and specificity (93.5%). Liang Wang (36) applied the same mass spectrometry technology as L. Wang (37) but constructed an ANN model based on five potential biomarkers. This model achieved sensitivity and specificity values of 91.7% and 90.0%, respectively. V. Janša (21) created an SVM model utilizing data from antibody microarrays and found an AUC of > 0.83, a sensitivity of 81%, and a specificity of 100%. Natalia Starodubtseva (17) employed the lipidome of menstrual blood to establish a diagnostic model using LR. Their results were an AUC of 0.87, a sensitivity of 81%, and a specificity of 85%. Liujing Huang (38) extracted gut, cervical mucus, and peritoneal fluid microflora. Then, the RF method was applied to achieve a sensitivity of 84.7% and a specificity of 80.6%.

## 4 Discussion

### 4.1 Summary of the main findings

This meta-analysis thoroughly evaluated the efficacy of ML and DL algorithms in detecting endometriosis. Initially, 2,380 studies were considered, and 45 reports were ultimately included after the screening process. Of those, 30 (67%) explored clinical characteristics as variables for diagnostic models, six (13%) focused on genetic regulators, three (7%) on imaging data, and six (13%) on other omics data. The results revealed that these approaches achieved relatively favorable AUCs, sensitivities, and specificities in identifying endometriosis from healthy women or those with similar symptoms. Additionally, our analysis indicated that MLMs based on genetic regulators displayed an even higher range of AUC, sensitivity, and specificity: 0.982 (95% CI: 0.975-0.990), 0.94 (95% CI: 0.90-0.97), and 0.99 (95% CI: 0.94-1.00), respectively, in the training set, supporting a previous study (39). These findings underscore the potential role of genetic regulators in endometriosis development.

### 4.2 Comparison with previous reviews

Previous systematic reviews highlighted the potential of AI in diagnosing endometriosis. Sivajohan et al.’s scoping review indicated that AI models utilizing diverse data types, including biomarkers, imaging, and clinical variables, achieved pooled sensitivity from 81.7% to 96.7% and specificity from 70.7% to 91.6%, suggesting robust diagnostic performance in controlled settings (3). Furthermore, current reviews emphasized significant methodological heterogeneity, noting variations in algorithms (e.g., LR, SVM, RF), diagnostic targets (e.g., ovarian endometriosis versus deep infiltrating endometriosis), and evaluation metrics, which impede direct comparison across studies (40). These reviews consistently identified a critical gap: the lack of quantitative synthesis to establish standardized diagnostic benchmarks. While prior syntheses offered valuable narrative insights, none conducted meta-analyses to derive pooled accuracy estimates. The present meta-analysis addresses this gap by systematically synthesizing existing evidence and providing pooled diagnostic accuracy estimates for ML-based endometriosis diagnostic models.

Feature selection played a central role in shaping the performance of the models included in the study. Input variables used for endometriosis diagnosis primarily consisted of clinical features, genetic regulators, radiomics, and other omics data. CA-125 levels, visual analog scale (VAS), history of dysmenorrhea, body mass index, and age were the most frequently used clinical inputs and formed the core of the interpretable models in the eligible studies. CA-125, a serum biomarker commonly used in diagnosing endometriosis, correlates with disease severity, particularly in advanced stages (41). Pain is widely regarded as a key diagnostic factor for endometriosis, and the VAS is the most commonly used tool for assessing it, demonstrating good validity, test-retest reliability, and consistency (42). Since clinical features are easily accessible during routine gynecological evaluations, they are practical for use in clinical practice, particularly in early population screening.

In contrast, an increasing number of studies have incorporated genetic regulators, primarily derived from miRNA datasets. miRNAs are believed to play a vital role in endometriosis pathogenesis by regulating inflammation, cell proliferation, angiogenesis, and tissue remodeling. In our study, models based on miRNA expression profiles achieved relatively high AUC values (>0.85), supporting the potential value of transcriptomic features in differential diagnosis. Prior systematic reviews have also reported the moderate to high diagnostic accuracy of circulating miRNAs in endometriosis. However, their clinical application has been limited by differences in sample size and detection platforms (43). Individual studies have reported promising results for models utilizing other omics, indicating the potential value of integrating molecular-level data. However, their heterogeneity and limited external validation reduce their general applicability. These findings should be interpreted with caution, as most studies lack standardized pipelines and reproducibility assessments.

In the field of imaging, research has primarily focused on related diseases, such as endometrial cancer, while paying relatively little attention to endometriosis. For instance, a systematic review by Lecointre et al. indicated that imaging-based methods for identifying endometrial cancer lack sufficient evidence and are in the early stages of development (29). High-quality prospective studies and reliable external validation are essential for advancing the clinical application of these methods. Our analysis suggests that, although multi-omics and imaging methods show great potential, models based on routinely available clinical data are the most practical and scalable for real-world diagnostic settings.

Furthermore, the type of ML model chosen determines the balance between accuracy and interpretability. The studies included in this research predominantly relied on LR, especially for clinical features. This implies that models combining clinical biomarkers prioritize interpretability over other types. However, our study found that ensemble and boosting algorithms, such as LightGBM, XGBoost, and AdaBoost, performed better. Except for the training set within the clinical features, the combined AUC, sensitivity, and specificity were higher than the metrics obtained using LR models alone in each feature type. Zhang et al. (30) reported consistent ML prediction results for gestational diabetes, which further supports the idea that some non-LR algorithms may offer higher diagnostic value, particularly in complex or high-dimensional datasets.

### 4.3 Advantages and limitations

This meta-analysis has several strengths. First, it covered a broad and systematic range of the most recent research findings. Second, the study was conducted and reported strictly according to established guidelines. Third, the quantitative evaluation, which was based on input feature grouping, revealed heterogeneity across studies and trends that could inform future research. However, this study also has certain limitations. First, endometriosis exhibits substantial phenotypic diversity, including superficial peritoneal lesions, ovarian endometriomas, and deep-infiltrating forms with varying disease severity. However, many of the included primary studies did not sufficiently explore the diagnostic performance of ML across these subtypes. This limits the interpretation of the diagnostic value of ML for specific endometriosis classifications. Future research should aim to improve endometriosis detection overall and develop robust multi-class ML models that can aid in subtype diagnosis. Second, the definitions of endometriosis cases and control groups varied across the included studies. Some studies relied on laparoscopic confirmation for diagnosis, while others used imaging or symptom-based criteria. Similarly, control groups ranged from healthy women to patients with symptoms but no confirmed diagnosis, and even included cases of non-endometriosis confirmed by surgery. The primary data did not sufficiently detail these criteria to allow for targeted subgroup analysis, which could introduce bias into the pooled diagnostic performance estimates and distort the overall accuracy assessment. Future studies should standardize the reporting of phenotypes, severity, diagnostic criteria, and control group definitions to facilitate stratified analyses in meta-analyses and enhance the precision and clinical applicability of results. Third, two studies (by Ulan Tore et al. and Krystian Zieliński et al.) exhibited severe class imbalance among the included literature. Imbalanced data can impact model development and pose challenges to model robustness (31). However, the number of such studies was limited, and their specific influence on the overall outcomes was not investigated further via subgroup analysis. Future research should employ strategies such as oversampling techniques to mitigate the effects of class imbalance on modeling results. Additionally, the limited number of ML modeling studies involving certain omics features and imaging data affected the robustness of the results. Lastly, most studies used retrospective designs and lacked independent validation, affecting the interpretation of our findings. We recommend that future research focus on refining the data standardization process to enhance the models’ external validity and clinical applicability.

## 5 Conclusions

ML demonstrates notable accuracy and application potential in diagnosing endometriosis. Predictive models built by integrating multimodal data, such as imaging and clinical indicators, excel at differentiating lesions from normal tissues and distinguishing between various lesion subtypes. This offers crucial technical support for early clinical diagnosis and precise subtyping. Nevertheless, existing studies still exhibit methodological limitations. Therefore, future research should pursue multicenter collaborations, establish standardized datasets, and implement external validation systems to comprehensively evaluate model stability and generalizability. Developing tools readily translatable to clinical practice will ultimately enable early screening, precise diagnosis, and personalized treatment for endometriosis, thereby improving patient outcomes.

## Data Availability

The original contributions presented in the study are included in the article/**Supplementary Material**. Further inquiries can be directed to the corresponding author.
