# Correction: Diagnostic accuracy of machine learning for endometriosis: a systematic review and meta-analysis

**DOI:** 10.3389/fendo.2026.1799754

**Published:** 2026-02-18

**Authors:** Bingyi Zhang, Xiaoli Lv, Dan Li, Longtao Zhang, Ziyang Ru, Yuxia Ma

**Affiliations:** School of Acupuncture and Tuina, Shandong University of Traditional Chinese Medicine, Jinan, Shandong, China

**Keywords:** diagnosis, endometriosis, machine learning, meta-analysis, systematic review

There were some errors in several reference list entries in the article:

Reference “32” was erroneously written as “Wölfler MM, Schwamborn K, Otten D, Hornung D, Liu H, Rath W. Mass spectrometry and serum pattern profiling for analyzing the individual risk for endometriosis: promising insights? *Fertil Steril*. (2009) 91:2331-7. doi: 10.1016/j.fertnstert.2008.03.064”. It should be “Miao K, Lv Q, Zhang L, Zhao N, Dong X. Discriminative diagnosis of ovarian endometriosis cysts and benign mucinous cystadenomas based on the ConvNeXt algorithm. *Eur J Obstet Gynecol Reprod Biol*. (2024) 298:135-139. doi: 10.1016/j.ejogrb.2024.05.010”.

Reference “33” was erroneously written as “Wang L, Zheng W, Mu L, Zhang SZ. Identifying biomarkers of endometriosis using serum protein fingerprinting and artificial neural networks. *Int J Gynaecol Obstet*. (2008) 101:253-8. doi: 10.1016/j.ijgo.2008.01.018”. It should be “Hu P, Gao Y, Zhang Y, Sun K. Ultrasound image-based deep learning to differentiate tubal-ovarian abscess from ovarian endometriosis cyst. *Front Physiol*. (2023) 14:1101810. doi: 10.3389/fphys.2023.1101810”.

Reference “34” was erroneously written as “Wang L, Liu HY, Shi HH, Lang JH, Sun W. Urine peptide patterns for non-invasive diagnosis of endometriosis: a preliminary prospective study. *Eur J Obstet Gynecol Reprod Biol*. (2014) 177:23-8. doi: 10.1016/j.ejogrb.2014.03.011”. It should be “Liu L, Cai W, Zhou C, Tian H, Wu B, Zhang J, et al. Ultrasound radiomics-based artificial intelligence model to assist in the differential diagnosis of ovarian endometrioma and ovarian dermoid cyst. *Front Med (Lausanne)*. (2024) 11:1362588. doi: 10.3389/fmed.2024.1362588”.

Reference “35” was erroneously written as “Huang L, Liu B, Liu Z, Feng W, Liu M, Wang Y, et al. Gut microbiota exceeds cervical microbiota for early diagnosis of endometriosis. *Front Cell Infect Microbiol*. (2021) 11:788836. doi: 10.3389/fcimb.2021.788836”. It should be “Wölfler MM, Schwamborn K, Otten D, Hornung D, Liu H, Rath W. Mass spectrometry and serum pattern profiling for analyzing the individual risk for endometriosis: promising insights? *Fertil Steril*. (2009) 91:2331-7. doi: 10.1016/j.fertnstert.2008.03.064”.

Reference “36” was erroneously written as “Zafari N, Bahramy A, Majidi Zolbin M, Emadi Allahyari S, Farazi E, Hassannejad Z, et al. microRNAs as novel diagnostic biomarkers in endometriosis patients: a systematic review and meta-analysis. *Expert Rev Mol Diagn*. (2022) 22:479-95. doi: 10.1080/14737159.2021.1960508”. It should be “Wang L, Zheng W, Mu L, Zhang SZ. Identifying biomarkers of endometriosis using serum protein fingerprinting and artificial neural networks. *Int J Gynaecol Obstet*. (2008) 101:253-8. doi: 10.1016/j.ijgo.2008.01.018”.

Reference “37” was erroneously written as “Etrusco A, Barra F, Chiantera V, Ferrero S, Bogliolo S, Evangelisti G, et al. Current medical therapy for adenomyosis: from bench to bedside. *Drugs*. (2023) 83:1595-611. doi: 10.1007/s40265-023-01957-7”. It should be “Wang L, Liu HY, Shi HH, Lang JH, Sun W. Urine peptide patterns for non-invasive diagnosis of endometriosis: a preliminary prospective study. *Eur J Obstet Gynecol Reprod Biol*. (2014) 177:23-8. doi: 10.1016/j.ejogrb.2014.03.011”.

Reference “38” was erroneously written as “Chen FP, Soong YK, Lee N, Lo SK. The use of serum CA-125 as a marker for endometriosis in patients with dysmenorrhea for monitoring therapy and for recurrence of endometriosis. *Acta Obstet Gynecol Scand*. (1998) 77:665-70. doi: 10.1034/j.1600-0412.1998.770615.x”. It should be “Huang L, Liu B, Liu Z, Feng W, Liu M, Wang Y, et al. Gut microbiota exceeds cervical microbiota for early diagnosis of endometriosis. *Front Cell Infect Microbiol*. (2021) 11:788836. doi: 10.3389/fcimb.2021.788836”.

Reference “39” was erroneously written as “Bourdel N, Alves J, Pickering G, Ramilo I, Roman H, Canis M. Systematic review of endometriosis pain assessment: how to choose a scale? *Hum Reprod Update*. (2015) 21:136-52. doi: 10.1093/humupd/dmu046”. It should be “Zafari N, Bahramy A, Majidi Zolbin M, Emadi Allahyari S, Farazi E, Hassannejad Z, et al. microRNAs as novel diagnostic biomarkers in endometriosis patients: a systematic review and meta-analysis. *Expert Rev Mol Diagn*. (2022) 22:479-95. doi: 10.1080/14737159.2021.1960508”.

Reference “40” was erroneously written as “Agrawal S, Tapmeier T, Rahmioglu N, Kirtley S, Zondervan K, Becker C. The miRNA mirage: how close are we to finding a non-invasive diagnostic biomarker in endometriosis? A Systematic Review. *Int J Mol Sci*. (2018) 19:599. doi: 10.3390/ijms19020599”. It should be “Etrusco A, Barra F, Chiantera V, Ferrero S, Bogliolo S, Evangelisti G, et al. Current medical therapy for adenomyosis: from bench to bedside. *Drugs*. (2023) 83:1595-611. doi: 10.1007/s40265-023-01957-7”.

Reference “41” was erroneously written as “Lecointre L, Dana J, Lodi M, Akladios C, Gallix B. Artificial intelligence-based radiomics models in endometrial cancer: A systematic review. *Eur J Surg Oncol*. (2021) 47:2734-41. doi: 10.1016/j.ejso.2021.06.023”. It should be “Chen FP, Soong YK, Lee N, Lo SK. The use of serum CA-125 as a marker for endometriosis in patients with dysmenorrhea for monitoring therapy and for recurrence of endometriosis. *Acta Obstet Gynecol Scand*. (1998) 77:665-70. doi: 10.1034/j.1600-0412.1998.770615.x”.

Reference “42” was erroneously written as “Zhang Z, Yang L, Han W, Wu Y, Zhang L, Gao C, et al. Machine learning prediction models for gestational diabetes mellitus: meta-analysis. *J Med Internet Res*. (2022) 24:e26634. doi: 10.2196/26634”. It should be “Bourdel N, Alves J, Pickering G, Ramilo I, Roman H, Canis M. Systematic review of endometriosis pain assessment: how to choose a scale? *Hum Reprod Update*. (2015) 21:136-52. doi: 10.1093/humupd/dmu046”.

Reference “43” was erroneously written as “Thölke P, Mantilla-Ramos YJ, Abdelhedi H, Maschke C, Dehgan A, Harel Y, et al. Class imbalance should not throw you off balance: Choosing the right classifiers and performance metrics for brain decoding with imbalanced data. *Neuroimage*. (2023) 277:120253. doi: 10.1016/j.neuroimage.2023.120253”. It should be “Agrawal S, Tapmeier T, Rahmioglu N, Kirtley S, Zondervan K, Becker C. The miRNA mirage: how close are we to finding a non-invasive diagnostic biomarker in endometriosis? A Systematic Review. *Int J Mol Sci*. (2018) 19:599. doi: 10.3390/ijms19020599”.

Reference “29” was erroneously written as “Miao K, Lv Q, Zhang L, Zhao N, Dong X. Discriminative diagnosis of ovarian endometriosis cysts and benign mucinous cystadenomas based on the ConvNeXt algorithm. *Eur J Obstet Gynecol Reprod Biol*. (2024) 298:135-139. doi: 10.1016/j.ejogrb.2024.05.010”. It should be “Lecointre L, Dana J, Lodi M, Akladios C, Gallix B. Artificial intelligence-based radiomics models in endometrial cancer: A systematic review. *Eur J Surg Oncol*. (2021) 47:2734-41. doi: 10.1016/j.ejso.2021.06.023”.

Reference “30” was erroneously written as “Hu P, Gao Y, Zhang Y, Sun K. Ultrasound image-based deep learning to differentiate tubal-ovarian abscess from ovarian endometriosis cyst. *Front Physiol*. (2023) 14:1101810. doi: 10.3389/fphys.2023.1101810”. It should be “Zhang Z, Yang L, Han W, Wu Y, Zhang L, Gao C, et al. Machine learning prediction models for gestational diabetes mellitus: meta-analysis. *J Med Internet Res*. (2022) 24:e26634. doi: 10.2196/26634”.

Reference “31” was erroneously written as “Liu L, Cai W, Zhou C, Tian H, Wu B, Zhang J, et al. Ultrasound radiomics-based artificial intelligence model to assist in the differential diagnosis of ovarian endometrioma and ovarian dermoid cyst. *Front Med (Lausanne)*. (2024) 11:1362588. doi: 10.3389/fmed.2024.1362588”. It should be “Thölke P, Mantilla-Ramos YJ, Abdelhedi H, Maschke C, Dehgan A, Harel Y, et al. Class imbalance should not throw you off balance: Choosing the right classifiers and performance metrics for brain decoding with imbalanced data. *Neuroimage*. (2023) 277:120253. doi: 10.1016/j.neuroimage.2023.120253”.

The original version of this article has been updated.

