# Supplementary material for: Diagnostic accuracy of machine learning for endometriosis: a systematic review and meta-analysis
Source: Front Endocrinol (Lausanne). 2026 Jan 27;16:1735567. doi: 10.3389/fendo.2025.1735567 (PMC12886017; doi:10.3389/fendo.2025.1735567)
Supplement: Supplementary file 1 [file DataSheet1.docx]

# Supplementary Materials

**Supplementary Table S1** Literature search strategy

Pubmed

| Search number | Query | Results |
| --- | --- | --- |
| #1 | "Endometriosis"[Mesh] | 26916 |
| #2 | ((((Endometriosis[Title/Abstract]) OR (Endometrioses[Title/Abstract])) OR (Endometrioma[Title/Abstract])) OR (Endometriomas[Title/Abstract])) OR (adenomyosis externa[Title/Abstract]) | 32,062 |
| #3 | ("Endometriosis"[Mesh]) OR (((((Endometriosis[Title/Abstract]) OR (Endometrioses[Title/Abstract])) OR (Endometrioma[Title/Abstract])) OR (Endometriomas[Title/Abstract])) OR (adenomyosis externa[Title/Abstract])) | 35,983 |
| #4 | Machine learning[MeSH Terms] | 76,776 |
| #5 | (((((((((((((((((((((((((((((((((((Machine learning[Title/Abstract]) OR (artificial intelligence[Title/Abstract])) OR (Transfer Learning[Title/Abstract])) OR (Deep learning[Title/Abstract])) OR (Ensemble Learning[Title/Abstract])) OR (prediction model[Title/Abstract])) OR (risk model[Title/Abstract])) OR (risk score[Title/Abstract])) OR (random forest[Title/Abstract])) OR (neural network[Title/Abstract])) OR (neural networks[Title/Abstract])) OR (CNN[Title/Abstract])) OR (K-Nearest Neighbor[Title/Abstract])) OR (Support vector machine[Title/Abstract])) OR (SVM[Title/Abstract])) OR (Gradient Boosting Machine[Title/Abstract])) OR (Nomogram[Title/Abstract])) OR (XGBoost[Title/Abstract])) OR (Adaboost[Title/Abstract])) OR (LightGBM[Title/Abstract])) OR (CatBoost[Title/Abstract])) OR (Gradient Boosting[Title/Abstract])) OR (Decision tree[Title/Abstract])) OR (Regression Trees[Title/Abstract])) OR (ResNet[Title/Abstract])) OR (AlexNet[Title/Abstract])) OR (VGGNet[Title/Abstract])) OR (GoogLeNet[Title/Abstract])) OR (Naive Bayesian[Title/Abstract])) OR (Multilayer perceptron[Title/Abstract])) OR (Bayesian network[Title/Abstract])) OR (Radiomics[Title/Abstract])) OR (Radiomic[Title/Abstract])) OR (radiomics-based[Title/Abstract])) OR (radiomic signature[Title/Abstract])) OR (Texture[Title/Abstract]) | 452,473 |
| #6 | (Machine learning[MeSH Terms]) OR ((((((((((((((((((((((((((((((((((((Machine learning[Title/Abstract]) OR (artificial intelligence[Title/Abstract])) OR (Transfer Learning[Title/Abstract])) OR (Deep learning[Title/Abstract])) OR (Ensemble Learning[Title/Abstract])) OR (prediction model[Title/Abstract])) OR (risk model[Title/Abstract])) OR (risk score[Title/Abstract])) OR (random forest[Title/Abstract])) OR (neural network[Title/Abstract])) OR (neural networks[Title/Abstract])) OR (CNN[Title/Abstract])) OR (K-Nearest Neighbor[Title/Abstract])) OR (Support vector machine[Title/Abstract])) OR (SVM[Title/Abstract])) OR (Gradient Boosting Machine[Title/Abstract])) OR (Nomogram[Title/Abstract])) OR (XGBoost[Title/Abstract])) OR (Adaboost[Title/Abstract])) OR (LightGBM[Title/Abstract])) OR (CatBoost[Title/Abstract])) OR (Gradient Boosting[Title/Abstract])) OR (Decision tree[Title/Abstract])) OR (Regression Trees[Title/Abstract])) OR (ResNet[Title/Abstract])) OR (AlexNet[Title/Abstract])) OR (VGGNet[Title/Abstract])) OR (GoogLeNet[Title/Abstract])) OR (Naive Bayesian[Title/Abstract])) OR (Multilayer perceptron[Title/Abstract])) OR (Bayesian network[Title/Abstract])) OR (Radiomics[Title/Abstract])) OR (Radiomic[Title/Abstract])) OR (radiomics-based[Title/Abstract])) OR (radiomic signature[Title/Abstract])) OR (Texture[Title/Abstract])) | 457,982 |
| #7 | (("Endometriosis"[Mesh]) OR (((((Endometriosis[Title/Abstract]) OR (Endometrioses[Title/Abstract])) OR (Endometrioma[Title/Abstract])) OR (Endometriomas[Title/Abstract])) OR (adenomyosis externa[Title/Abstract]))) AND ((Machine learning[MeSH Terms]) OR ((((((((((((((((((((((((((((((((((((Machine learning[Title/Abstract]) OR (artificial intelligence[Title/Abstract])) OR (Transfer Learning[Title/Abstract])) OR (Deep learning[Title/Abstract])) OR (Ensemble Learning[Title/Abstract])) OR (prediction model[Title/Abstract])) OR (risk model[Title/Abstract])) OR (risk score[Title/Abstract])) OR (random forest[Title/Abstract])) OR (neural network[Title/Abstract])) OR (neural networks[Title/Abstract])) OR (CNN[Title/Abstract])) OR (K-Nearest Neighbor[Title/Abstract])) OR (Support vector machine[Title/Abstract])) OR (SVM[Title/Abstract])) OR (Gradient Boosting Machine[Title/Abstract])) OR (Nomogram[Title/Abstract])) OR (XGBoost[Title/Abstract])) OR (Adaboost[Title/Abstract])) OR (LightGBM[Title/Abstract])) OR (CatBoost[Title/Abstract])) OR (Gradient Boosting[Title/Abstract])) OR (Decision tree[Title/Abstract])) OR (Regression Trees[Title/Abstract])) OR (ResNet[Title/Abstract])) OR (AlexNet[Title/Abstract])) OR (VGGNet[Title/Abstract])) OR (GoogLeNet[Title/Abstract])) OR (Naive Bayesian[Title/Abstract])) OR (Multilayer perceptron[Title/Abstract])) OR (Bayesian network[Title/Abstract])) OR (Radiomics[Title/Abstract])) OR (Radiomic[Title/Abstract])) OR (radiomics-based[Title/Abstract])) OR (radiomic signature[Title/Abstract])) OR (Texture[Title/Abstract]))) | 265 |

Cochrane

| Search number | Query | Results |
| --- | --- | --- |
| #1 | MeSH descriptor: [Endometriosis] explode all trees | 1287 |
| #2 | (Endometriosis):ti,ab,kw OR (Endometrioses):ti,ab,kw OR (Endometrioma):ti,ab,kw OR (Endometriomas):ti,ab,kw OR (adenomyosis externa):ti,ab,kw | 3411 |
| #3 | #1 or #2 | 3411 |
| #4 | MeSH descriptor: [Machine Learning] explode all trees | 1023 |
| #5 | (Machine learning):ti,ab,kw OR (artificial intelligence):ti,ab,kw OR (Transfer Learning):ti,ab,kw OR (Deep learning):ti,ab,kw OR (Ensemble Learning):ti,ab,kw | 7899 |
| #6 | (prediction model):ti,ab,kw OR (risk model):ti,ab,kw OR (risk score):ti,ab,kw OR (random forest):ti,ab,kw OR (neural network):ti,ab,kw | 75366 |
| #7 | (neural networks):ti,ab,kw OR (CNN):ti,ab,kw OR (K-Nearest Neighbor):ti,ab,kw OR (Support vector machine):ti,ab,kw OR (SVM):ti,ab,kw | 2729 |
| #8 | (Gradient Boosting Machine):ti,ab,kw OR (Nomogram):ti,ab,kw OR (XGBoost):ti,ab,kw OR (Adaboost):ti,ab,kw OR (LightGBM):ti,ab,kw | 2121 |
| #9 | (CatBoost):ti,ab,kw OR (Gradient Boosting):ti,ab,kw OR (Decision tree):ti,ab,kw OR (Regression Trees):ti,ab,kw OR (ResNet):ti,ab,kw | 1356 |
| #10 | (AlexNet):ti,ab,kw OR (VGGNet):ti,ab,kw OR (GoogLeNet):ti,ab,kw OR (Naive Bayesian):ti,ab,kw OR (Multilayer perceptron):ti,ab,kw | 210 |
| #11 | (Bayesian network):ti,ab,kw OR (Radiomics):ti,ab,kw OR (Radiomic):ti,ab,kw | 1147 |
| #12 | (radiomics-based):ti,ab,kw OR (radiomic signature):ti,ab,kw OR (Texture):ti,ab,kw | 2376 |
| #13 | #4 or #5 or #6 or #7 or #8 or #9 or #10 or #11 or #12 | 85707 |
| #14 | #3 and #13 | 99 |

Embase

| Search number | Query | Results |
| --- | --- | --- |
| #1 | 'endometriosis'/exp | 52732 |
| #2 | endometriosis:ab,ti OR endometrioses:ab,ti OR endometrioma:ab,ti OR endometriomas:ab,ti OR 'adenomyosis externa':ab,ti | 46780 |
| #3 | #1 OR #2 | 57878 |
| #4 | 'machine learning'/exp | 515671 |
| #5 | 'machine learning':ab,ti OR 'artificial intelligence':ab,ti OR 'transfer learning':ab,ti OR 'deep learning':ab,ti OR 'ensemble learning':ab,ti OR 'prediction model':ab,ti OR 'risk model':ab,ti OR 'risk score':ab,ti OR 'random forest':ab,ti OR 'neural network':ab,ti OR 'neural networks':ab,ti OR cnn:ab,ti OR 'k-nearest neighbor':ab,ti OR 'support vector machine':ab,ti OR svm:ab,ti OR 'gradient boosting machine':ab,ti OR nomogram:ab,ti OR xgboost:ab,ti OR adaboost:ab,ti OR lightgbm:ab,ti OR catboost:ab,ti OR 'gradient boosting':ab,ti OR 'decision tree':ab,ti OR 'regression trees':ab,ti OR resnet:ab,ti OR alexnet:ab,ti OR vggnet:ab,ti OR googlenet:ab,ti OR 'naive bayesian':ab,ti OR 'multilayer perceptron':ab,ti OR 'bayesian network':ab,ti OR radiomics:ab,ti OR radiomic:ab,ti OR 'radiomics based':ab,ti OR 'radiomic signature':ab,ti OR texture:ab,ti | 587164 |
| #6 | #4 OR #5 | 831713 |
| #7 | #3 AND #6 | 672 |

Web of science

| Search number | Query | Results |
| --- | --- | --- |
| #1 | Endometriosis (Topic) OR Endometrioses (Topic) OR Endometrioma (Topic) OR Endometriomas (Topic) OR adenomyosis externa (Topic) | 31169 |
| #2 | Machine learning (Topic) OR artificial intelligence (Topic) OR Transfer Learning (Topic) OR Deep learning (Topic) OR Ensemble Learning (Topic) OR prediction model (Topic) OR risk model (Topic) OR risk score (Topic) OR random forest (Topic) OR neural network (Topic) OR neural networks (Topic) OR CNN (Topic) OR K-Nearest Neighbor (Topic) OR Support vector machine (Topic) OR SVM (Topic) OR Gradient Boosting Machine (Topic) OR Nomogram (Topic) OR XGBoost (Topic) OR Adaboost (Topic) OR LightGBM (Topic) OR CatBoost (Topic) OR Gradient Boosting (Topic) OR Decision tree (Topic) OR Regression Trees (Topic) OR ResNet (Topic) OR AlexNet (Topic) OR VGGNet (Topic) OR GoogLeNet (Topic) OR Naive Bayesian (Topic) OR Multilayer perceptron (Topic) OR Bayesian network (Topic) OR Radiomics (Topic) OR Radiomic (Topic) OR radiomics-based (Topic) OR radiomic signature (Topic) OR Texture (Topic) | 2912912 |
| #3 | #1 OR #2 | 1344 |

**Supplementary** **Table S2** Basic characteristics of eligible studies.

| No. | Author | DOI | Publication Year | Country | Patient Source | Endometriosis Cases | Total Cases | Cases in Training Set | Validation Set Generation Methods | Cases in Validation Set | Model Types Used | Modeling Variables |
| --- | --- | --- | --- | --- | --- | --- | --- | --- | --- | --- | --- | --- |
| 1 | Ningning Zhao | [10.1186/s12905-024-03334-2](https://doi.org/10.1186/s12905-024-03334-2) | 2024 | China | Single-center | 106 | 309 | 200 | Random sampling | 87 | RF, SVM, NB, LR, LogitBoost, DT, neural networks | Clinical variables |
| 2 | Krystian Zieliński | [10.1186/s12958-023-01156-9](https://doi.org/10.1186/s12958-023-01156-9) | 2023 | Poland | Invicta database (retrospective database) | 1236 | 11,819 | —— | 5-fold cross-validation | —— | LightGBM | Clinical variables |
| 3 | He Zhao | [10.1080/01443615.2023.2188072](https://doi.org/10.1080/01443615.2023.2188072) | 2023 | China | Single-center retrospective data | 280 | 280 | 140 ( stage 1-4) | Equally divided by year | 140 | LR | Clinical variables |
| 4 | Jie Zhang | [10.1016/j.jmig.2023.12.008](https://doi.org/10.1016/j.jmig.2023.12.008) | 2023 | China | Single-center retrospective data | 899 | 1365 | 910 | Random sampling | 455 | LR | Clinical variables |
| 5 | Yanhua Yang | [10.3390/diagnostics12102348](https://doi.org/10.3390/diagnostics12102348) | 2022 | China | Single-center prospective database | 326 (severe) | 491 (confirmed) | —— | —— | —— | LR | Clinical variables |
| 6 | [Monika M. Wölfler](https://pubmed.ncbi.nlm.nih.gov/?term=W%C3%B6lfler+MM&cauthor_id=18555242) | [10.1016/j.fertnstert.2008.03.064](https://doi.org/10.1016/j.fertnstert.2008.03.064) | 2009 | Germany | Single-center retrospective data | 51 | 90 | 90 | —— | —— | DTA, GA | Clinical variables |
| 7 | [M.M. Wölfler](https://pubmed.ncbi.nlm.nih.gov/?term=W%C3%B6lfler+MM&cauthor_id=15760962) | [10.1093/humrep/deh796](https://doi.org/10.1093/humrep/deh796) | 2005 | Germany | Single-center retrospective data | 25 | 48 | 48 | —— | —— | LR | Clinical variables |
| 8 | Weiying Wang | [10.3389/fdgth.2024.1463419](https://doi.org/10.3389/fdgth.2024.1463419) | 2024 | China | Single-center retrospective database | 329 | 558 (EMs/AD) | 80% | 5-fold cross-validation | 20% | LR, XGBoost, MLP, SVM, RF | Clinical variables |
| 9 | Liang Wang | [10.1016/j.ijgo.2008.01.018](https://doi.org/10.1016/j.ijgo.2008.01.018) | 2008 | China | Single-center retrospective data | 36 | 66 | 44 | Random sampling | 10 | ANN | Clinical variables |
| 10 | L. Wang | 10.1016/j.ejogrb.2014.03.011 | 2014 | China | Single-center retrospective data | 60 | 122 | 122 | External validation | 25 (separately sampled) | GA, DTA, QC | Clinical variables |
| 11 | A. Vodolazkaia | 10.1093/humrep/des234 | 2012 | Belgium | Single-center retrospective data | 232 | 353 | 235 | Stratified random sampling | 118 | LR, LS-SVM | Clinical variables |
| 12 | Nina Julie Verket | 10.1136/bmjopen-2019-030346 | 2019 | Norway | Single-center cross-sectional data | 157 | 313 | 313 | —— | —— | LG, LASSO | Clinical variables |
| 13 | Ulan Tore | [10.3390/biomedicines11113015](https://doi.org/10.3390/biomedicines11113015) | 2023 | Kazakhstan | Multi-center retrospective database | 5145 | 612777 | 490221 | Stratified random sampling | 122556 | LR, DTA, RF, AdaBoost, XGBoost | Clinical variables |
| 14 | Maria Szubert | [10.3390/medicina59030499](https://doi.org/10.3390/medicina59030499) | 2023 | Poland | Single-center retrospective data | 71 | 101 | 71 | Random sampling | 30 | LASSO, LR | Clinical variables |
| 15 | Barbara J. Stegmann | 10.1016/j.fertnstert.2007.11.038 | 2009 | United States | Single-center retrospective data | 320 | 487 | 334 | Random sampling | 153 | LR | Clinical variables |
| 16 | Natalia Starodubtseva | [10.3390/biom14080899](https://doi.org/10.3390/biom14080899) | 2024 | Russia | Single-center retrospective data | 23 | 39 | —— | —— | —— | LR | Clinical variables |
| 17 | Kelechi E. Nnoaham | [10.1016/j.fertnstert.2012.04.022](https://doi.org/10.1016/j.fertnstert.2012.04.022) | 2012 | Thirteen countries including the United Kingdom | Multi-center retrospective data | 724 (Phase 1: 360 cases, Phase 2: 364 cases) | 1,396 (Phase 1: 771 cases, Phase 2: 625 cases) | 771 (Phase 1) | External validation (prospective, multicenter, phase 2 data as validation set) | 625 (Phase 2) | LR | Clinical variables |
| 18 | Sarah Moustafa | [10.1016/j.ajog.2020.02.050](https://doi.org/10.1016/j.ajog.2020.02.050) | 2020 | United States | Single-center retrospective data | 41 | 100 | 100 | External validation (independent dataset) | 48 | RF | Genetic characteristics |
| 19 | A. Mihalyi | [10.1093/humrep/dep425](https://doi.org/10.1093/humrep/dep425) | 2010 | Belgium | Single-center retrospective data | 201 | 294 | —— | —— | —— | LSSVM, LR | Clinical variables |
| 20 | Kuo Miao | [10.1016/j.ejogrb.2024.05.010](https://doi.org/10.1016/j.ejogrb.2024.05.010) | 2024 | China | Single-center retrospective data | —— | —— | —— | Random sampling | —— | ConvNeXt | Radiomics |
| 21 | Lu Liu | [10.3389/fmed.2024.1362588](https://doi.org/10.3389/fmed.2024.1362588) | 2024 | China | Single-center retrospective data | —— | —— | 326 | Random sampling | 81 | LR, SVM, KNN, LightGBM, MLP | Clinical variables, radiomics |
| 22 | M.C. Lafay Pillet | [10.1093/humrep/deu128](https://doi.org/10.1093/humrep/deu128) | 2014 | France | Single-center retrospective data | 326 (including 164 cases with DIE) | 326 | 211 | Random sampling (randomly select two-thirds of patients as training set, remaining one-third as validation set) | 115 | LR | Clinical variables (e.g., pain-related variables, infertility, medical history, etc.) |
| 23 | Lutz Konrad | [10.3390/jcm12134231](https://doi.org/10.3390/jcm12134231) | 2023 | Germany | Single-center retrospective data | 177 | 269 | 269 | —— | —— | CART | Clinical variables |
| 24 | Liujing Huang | [10.3389/fcimb.2021.788836](https://doi.org/10.3389/fcimb.2021.788836) | 2021 | China | Single-center retrospective data | 21 | 41 | —— | Internal validation (5-fold cross-validation) | —— | RF | Clinical variables |
| 25 | Bárbara Herranz - Blanco | [10.3390/biom13071052](https://doi.org/10.3390/biom13071052) | 2023 | Spain | Single-center retrospective database | 136 (Study 1), 52 (Study 2) | 204 (Study 1), 79 (Study 2) | 204 (Study 1) | External validation (Study 2 uses an independent sample cohort) | 79 (Study 2) | LR | Serum biomarkers (CA125, BDNF), clinical variables (history of prior surgeries, dysmenorrhea symptoms, dysmenorrhea severity, age at first analgesic use, age at first ovarian cyst diagnosis, age at first dyspareunia, etc.) |
| 26 | Zaixin Guo | [10.3389/fmed.2020.570483](https://doi.org/10.3389/fmed.2020.570483) | 2020 | China | Single-center retrospective data | 573 (Training set: 377, Validation set: 196) | 1016 | 667 | Internal validation (random sampling) | 339 | LR | Clinical variables |
| 27 | Stefano Guerriero | [10.1016/j.ejogrb.2021.04.012](https://doi.org/10.1016/j.ejogrb.2021.04.012) | 2021 | Italy, Spain | Single-center retrospective data | 106 | 333 | 222 | Random sampling | 110 | k-NN, NB, NN, SVM, DT, RF, LR | Clinical variables |
| 28 | Anat Goldstein | [10.1038/s41598-023-32761-8](https://doi.org/10.1038/s41598-023-32761-8) | 2023 | Israel | Single-center retrospective data | 474 | 886 | —— | 10-fold cross-validation | —— | DT, RF, GBC, AdaBoost | Clinical variables |
| 29 | [Danièle Gagné](https://pubmed.ncbi.nlm.nih.gov/?term=Gagn%C3%A9+D&cauthor_id=14556806) | [10.1016/s0015-0282(03)01153-1](https://doi.org/10.1016/s0015-0282(03)01153-1) | 2003 | Canada | Multi-center retrospective data | 173 | 368 | —— | Bootstrapping (internal validation) | —— | LR | Clinical variables |
| 30 | Arnaud Fauconnier | [10.1016/j.fertnstert.2021.07.1205](https://doi.org/10.1016/j.fertnstert.2021.07.1205) | 2021 | Netherlands | Multi-center retrospective data | 105 | 302 | —— | —— | —— | LR | Clinical variables |
| 31 | Ting Chen | [10.1002/jcla.23947](https://doi.org/10.1002/jcla.23947) | 2021 | China | Single-center retrospective data | 137 | 274 | —— | —— | —— | LR | Clinical variables |
| 32 | Charles Chapron | [10.1016/j.eclinm.2021.101263](https://doi.org/10.1016/j.eclinm.2021.101263) | 2022 | France | Single-center retrospective data | 1195 | 2527 | 1685 | Random sampling | 842 | LR | Clinical variables |
| 33 | C.Chapron | [10.1093/humrep/deh627](https://doi.org/10.1093/humrep/deh627) | 2015 | France | Single-center retrospective data | 51 | 134 | —— | —— | —— | LR | Clinical variables |
| 34 | C.Calhaz-Jorge | [10.1093/humrep/deh374](https://doi.org/10.1093/humrep/deh374) | 2004 | Portugal | Single-center retrospective data | 488 | 1079 | —— | —— | —— | Logistic regression | Clinical variables |
| 35 | Sofiane Bendifallah | [10.3390/jcm11030612](https://doi.org/10.3390/jcm11030612) | 2022 | France | Single-center | 153 | 200 | —— | Internal validation (random sampling) | —— | RF | Genetic characteristics (miRNA) |
| 36 | Sofiane Bendifallah | [10.1038/s41598-021-04637-2](https://doi.org/10.1038/s41598-021-04637-2) | 2022 | France | Multi-center | 1,213 (Training set [1,126] + Validation set [87]) | 1,834 (Training set [1,734] + Validation set [100]) | 1734 | External validation (labeled as prospective) | 100 | LR, RF, DT, XGB, Voting Classifier (soft), Voting Classifier (hard) | Clinical variables |
| 37 | Sofiane Bendifallah | [10.1016/j.ejogrb.2024.04.020](https://doi.org/10.1016/j.ejogrb.2024.04.020) | 2024 | France | Potentially multi-center | —— | —— | —— | Internal validation | —— | —— | Genetic characteristics |
| 38 | Sofiane Bendifallah | [10.1056/EVIDoa2200282](https://doi.org/10.1056/evidoa2200282) | 2023 | France | Multi-center | 159 | 200 | —— | External validation (labeled as prospective, multicenter) | 200 | RF | Genetic characteristics |
| 39 | Sofiane Bendifallah | [10.1038/s41598-022-07771-7](https://doi.org/10.1038/s41598-022-07771-7) | 2022 | France | Single-center | 153 | 200 | —— | Internal validation (random sampling) | —— | RF, XGBoost, AdaBoost, LR | Genetic characteristics (miRNA) |
| 40 | Paula Brady | [10.3389/frph.2024.1360417](https://doi.org/10.3389/frph.2024.1360417) | 2024 | United States, etc. | Multi-center | 64 | 182 | 108 | Internal validation (randomly split internal replication phase samples into training and test sets at a 2:1 ratio) | 54 | NN | Genetic characteristics (miRNA expression), clinical variables (age, BMI, hormone use, pain symptoms, etc.) |
| 41 | Yu Dai | [10.1186/s12889-024-17825-2](https://doi.org/10.1186/s12889-024-17825-2) | 2024 | China | Single-center | 641 | 1287 | —— | External validation (single-center) | 400 | LR, LASSO | Clinical variables |
| 42 | S. Guerriero | [10.1002/uog.20289](https://doi.org/10.1002/uog.20289) | 2020 | Italy | Single-center | 106 | 333 | —— | —— | —— | Logistic regression | Clinical variables (ultrasound soft markers) |
| 43 | Ping Hu | [10.3389/fphys.2023.1101810](https://doi.org/10.3389/fphys.2023.1101810) | 2023 | China | Single-center retrospective data | 110 (OEC) | 188 (TOA + OEC) | 157 (TOA + OEC) | Internal + External Validation | 31 (TOA + OEC) | CNN | Radiomics (ultrasound imaging) |
| 44 | V Janša | [10.1038/s41598-021-00299-2](https://doi.org/10.1038/s41598-021-00299-2) | 2021 | Slovenia | Single-center retrospective data | 32 | 58 | 12 | —— | 46 | SVM | Peritoneal fluid |
| 45 | Giuseppe Ricci | [10.1371/journal.pone.0230828](https://doi.org/10.1371/journal.pone.0230828) | 2020 | Italy | Single-center | 51 | 151 | 151 | —— | —— | LR | Clinical variables |

**Supplementary Table S3 The AUC values of machine learning models for the diagnosis of endometriosis in the training and validation sets of the included studies.**

| No | Author | Year | Study | events | samplesize | Model | Variable | C-index(Roc) | SE | low | up |
| --- | --- | --- | --- | --- | --- | --- | --- | --- | --- | --- | --- |
| 1 | V Janša | 2021 | V Janša (2021) | 20 | 26 | SVM | Omics | 0.8330 | 0.062 | 0.712 | 0.954 |
| 2 | Paula Brady | 2024 | Paula Brady (2024) | 18 | 36 | ANN | Genetic | 0.7700 | 0.069 | 0.636 | 0.904 |
| 3 | Sarah Moustafa | 2020 | Sarah Moustafa (2020) | 24 | 48 | RF | Genetic | 0.9390 | 0.032 | 0.877 | 1.001 |
| 4 | Sofiane Bendifallah | 2022 | Sofiane Bendifallah (2022) | 87 | 100 | DT | Clinical | 0.7800 | 0.034 | 0.714 | 0.846 |
| 5 | Krystian Zieliński | 2023 | Krystian Zieliński (2023) | 1236 | 11819 | LightGBM | Clinical | 0.8200 | 0.006 | 0.808 | 0.832 |
| 6 | Lu Liu | 2024 | Lu Liu (2024) | 40 | 81 | LightGBM | Clinical | 0.5940 | 0.055 | 0.514 | 0.674 |
| 7 | He Zhao | 2023 | He Zhao (2023) | 68 | 140 | LR | Clinical | 0.7600 | 0.035 | 0.677 | 0.842 |
| 8 | Jie Zhang | 2023 | Jie Zhang (2023) | 312 | 455 | LR | Clinical | 0.8500 | 0.014 | 0.800 | 0.890 |
| 9 | Weiying Wang | 2024 | Weiying Wang (2024) | 66 | 112 | LR | Clinical | 0.7350 | 0.039 | 0.640 | 0.831 |
| 10 | A. Vodolazkaia | 2012 | A. Vodolazkaia (2012) | 77 | 118 | LR | Clinical | 0.7800 | 0.034 | 0.714 | 0.846 |
| 11 | A. Vodolazkaia | 2012 | A. Vodolazkaia (2012) | 77 | 118 | LR | Clinical | 0.7800 | 0.034 | 0.714 | 0.846 |
| 12 | Szubert | 2023 | Szubert (2023) | 25 | 30 | LR | Clinical | 0.8400 | 0.055 | 0.733 | 0.947 |
| 13 | Barbara J. Stegmann | 2009 | Barbara J. Stegmann (2009) | 101 | 153 | LR | Clinical | 0.6900 | 0.034 | 0.624 | 0.756 |
| 14 | Kelechi E. Nnoaham | 2012 | Kelechi E. Nnoaham (2012) | 364 | 625 | LR | Clinical | 0.8490 | 0.013 | 0.814 | 0.880 |
| 15 | M.C. Lafay Pillet | 2014 | M.C. Lafay Pillet (2014) | 63 | 115 | LR | Clinical | 0.8362 | 0.032 | 0.760 | 0.910 |
| 16 | Bárbara Herranz - Blanco | 2023 | Bárbara Herranz – Blanco (2023) | 52 | 77 | LR | Clinical | 0.7580 | 0.043 | 0.650 | 0.867 |
| 17 | Zaixin Guo | 2020 | Zaixin Guo (2020) | 64 | 339 | LR | Clinical | 0.7930 | 0.031 | 0.725 | 0.853 |
| 18 | Charles Chapron | 2022 | Charles Chapron (2022) | 395 | 842 | LR | Clinical | 0.7300 | 0.015 | 0.670 | 0.790 |
| 19 | Charles Chapron | 2022 | Charles Chapron (2022) | 395 | 842 | LR | Clinical | 0.7300 | 0.015 | 0.670 | 0.790 |
| 20 | Charles Chapron | 2022 | Charles Chapron (2022) | 220 | 308 | LR | Clinical | 0.7400 | 0.022 | 0.710 | 0.770 |
| 21 | Charles Chapron | 2022 | Charles Chapron (2022) | 220 | 308 | LR | Clinical | 0.8100 | 0.019 | 0.780 | 0.840 |
| 22 | Sofiane Bendifallah | 2022 | Sofiane Bendifallah (2022) | 87 | 100 | LR | Clinical | 0.8800 | 0.026 | 0.830 | 0.930 |
| 23 | Yu Dai | 2024 | Yu Dai (2024) | 92 | 260 | LR | Clinical | 0.7940 | 0.027 | 0.741 | 0.847 |
| 24 | A. Vodolazkaia | 2012 | A. Vodolazkaia (2012) | 77 | 118 | SVM | Clinical | 0.8100 | 0.032 | 0.748 | 0.872 |
| 25 | A. Vodolazkaia | 2012 | A. Vodolazkaia (2012) | 77 | 118 | SVM | Clinical | 0.8400 | 0.029 | 0.782 | 0.898 |
| 26 | Weiying Wang | 2024 | Weiying Wang (2024) | 66 | 112 | ANN | Clinical | 0.7440 | 0.038 | 0.650 | 0.839 |
| 27 | Weiying Wang | 2024 | Weiying Wang (2024) | 66 | 112 | RF | Clinical | 0.7310 | 0.039 | 0.634 | 0.827 |
| 28 | Sofiane Bendifallah | 2022 | Sofiane Bendifallah (2022) | 87 | 100 | RF | Clinical | 0.9200 | 0.021 | 0.879 | 0.961 |
| 29 | Weiying Wang | 2024 | Weiying Wang (2024) | 66 | 112 | SVM | Clinical | 0.7270 | 0.039 | 0.631 | 0.824 |
| 30 | Sofiane Bendifallah | 2022 | Sofiane Bendifallah (2022) | 87 | 100 | Voter classifier | Clinical | 0.9200 | 0.021 | 0.879 | 0.961 |
| 31 | Sofiane Bendifallah | 2022 | Sofiane Bendifallah (2022) | 87 | 100 | Voter classifier | Clinical | 0.9000 | 0.023 | 0.854 | 0.946 |
| 32 | Weiying Wang | 2024 | Weiying Wang (2024) | 66 | 112 | XGBoost | Clinical | 0.7470 | 0.038 | 0.652 | 0.842 |
| 33 | Tore | 2023 | Tore (2023) | 1029 | 122566 | XGBoost | Clinical | 0.7250 | 0.008 | 0.710 | 0.740 |
| 34 | Sofiane Bendifallah | 2022 | Sofiane Bendifallah (2022) | 87 | 100 | XGBoost | Clinical | 0.9300 | 0.020 | 0.891 | 0.969 |
| 35 | Lu Liu | 2024 | Lu Liu (2024) | 40 | 81 | LightGBM | Imaging | 0.9870 | 0.011 | 0.967 | 1.000 |
| 36 | Ping Hu | 2023 | Ping Hu (2023) | 21 | 31 | DL | Imaging | 0.9860 | 0.017 | 0.954 | 1.000 |
| 37 | Ping Hu | 2023 | Ping Hu (2023) | 10 | 14 | DL | Imaging | 1.0000 | 0.000 | 1.000 | 1.000 |
| 38 | Lu Liu | 2024 | Lu Liu (2024) | 40 | 81 | LightGBM | Imaging | 0.9670 | 0.018 | 0.940 | 0.995 |

**Supplementary Table S4 The diagnostic 2×2 contingency tables of machine learning models for the diagnosis of endometriosis in the training and validation sets of the included studies.**

| No | Author | Year | Study | Events | Sample size | Model | Variable | Sensitivity/Recall | Specificit | tp | fp | fn | tn |
| --- | --- | --- | --- | --- | --- | --- | --- | --- | --- | --- | --- | --- | --- |
| 1 | Krystian Zieliński | 2023 | Krystian Zieliński (2023) | 1236 | 11819 | LightGBM | Clinical | 0.7300 | 0.7600 | 902 | 2540 | 334 | 8043 |
| 2 | He Zhao | 2023 | He Zhao (2023) | 68 | 140 | LR | Clinical | 0.8720 | 0.7100 | 59 | 21 | 9 | 51 |
| 3 | Jie Zhang | 2023 | Jie Zhang (2023) | 312 | 455 | LR | Clinical | 0.9330 | 0.6640 | 291 | 48 | 21 | 95 |
| 4 | Weiying Wang | 2024 | Weiying Wang (2024) | 66 | 112 | LR | Clinical | 0.6840 | 0.7100 | 45 | 13 | 21 | 33 |
| 5 | Weiying Wang | 2024 | Weiying Wang (2024) | 66 | 112 | XGBoost | Clinical | 0.8030 | 0.6520 | 53 | 16 | 13 | 30 |
| 6 | Weiying Wang | 2024 | Weiying Wang (2024) | 66 | 112 | MLP | Clinical | 0.6580 | 0.7700 | 43 | 11 | 23 | 35 |
| 7 | Weiying Wang | 2024 | Weiying Wang (2024) | 66 | 112 | SVM | Clinical | 0.6990 | 0.6880 | 46 | 14 | 20 | 32 |
| 8 | Weiying Wang | 2024 | Weiying Wang (2024) | 66 | 112 | RF | Clinical | 0.6650 | 0.7410 | 44 | 12 | 22 | 34 |
| 9 | Liang Wang | 2007 | Liang Wang (2007) | 12 | 22 | ANN | Omics | 0.9170 | 0.9000 | 11 | 1 | 1 | 9 |
| 10 | A. Vodolazkaia | 2012 | A. Vodolazkaia (2012) | 77 | 118 | LR | Clinical | 0.8200 | 0.7500 | 63 | 10 | 14 | 31 |
| 11 | A. Vodolazkaia | 2012 | A. Vodolazkaia (2012) | 77 | 118 | LS-SVM | Clinical | 0.8200 | 0.6300 | 63 | 15 | 14 | 26 |
| 12 | A. Vodolazkaia | 2012 | A. Vodolazkaia (2012) | 77 | 118 | LR | Clinical | 0.8200 | 0.7500 | 63 | 10 | 14 | 31 |
| 13 | A. Vodolazkaia | 2012 | A. Vodolazkaia (2012) | 77 | 118 | LS-SVM | Clinical | 0.8200 | 0.7500 | 63 | 10 | 14 | 31 |
| 14 | Tore | 2023 | Tore (2023) | 1029 | 125513 | XGBoost | Clinical | 0.6860 | 0.6290 | 706 | 45087 | 323 | 76440 |
| 15 | Szubert | 2023 | Szubert (2023) | 25 | 30 | LR | Clinical | 0.8800 | 0.8000 | 22 | 1 | 3 | 4 |
| 16 | Kelechi E. Nnoaham | 2012 | Kelechi E. Nnoaham (2012) | 364 | 625 | LR | Clinical | 0.8226 | 0.7576 | 299 | 63 | 65 | 198 |
| 17 | Sarah Moustafa | 2020 | Sarah Moustafa (2020) | 24 | 48 | RF | Genetic | 0.8300 | 0.9600 | 20 | 1 | 4 | 23 |
| 18 | Lu Liu | 2024 | Lu Liu (2024) | 40 | 81 | LightGBM | Clinical | 0.4850 | 0.7980 | 19 | 8 | 21 | 33 |
| 19 | Lu Liu | 2024 | Lu Liu (2024) | 40 | 81 | LightGBM | Imaging | 0.8790 | 0.9710 | 35 | 1 | 5 | 40 |
| 20 | Lu Liu | 2024 | Lu Liu (2024) | 40 | 81 | LightGBM | Imaging | 0.8800 | 1.0000 | 35 | 0 | 5 | 41 |
| 21 | M.C. Lafay Pillet | 2014 | M.C. Lafay Pillet (2014) | 63 | 115 | LR | Clinical | 0.7622 | 0.7682 | 48 | 12 | 15 | 40 |
| 22 | Bárbara Herranz - Blanco | 2023 | Bárbara Herranz – Blanco (2023) | 52 | 77 | LR | Clinical | 0.4620 | 1.0000 | 24 | 0 | 28 | 25 |
| 23 | Zaixin Guo | 2020 | Zaixin Guo (2020) | 64 | 339 | LR | Clinical | 0.6870 | 0.7944 | 44 | 57 | 20 | 218 |
| 24 | Charles Chapron | 2022 | Charles Chapron (2022) | 395 | 842 | LR | Clinical | 0.7562 | 0.6028 | 299 | 178 | 96 | 269 |
| 25 | Charles Chapron | 2022 | Charles Chapron (2022) | 395 | 842 | LR | Clinical | 0.6284 | 0.7389 | 248 | 117 | 147 | 330 |
| 26 | Charles Chapron | 2022 | Charles Chapron (2022) | 220 | 308 | LR | Clinical | 0.7363 | 0.7194 | 162 | 25 | 58 | 63 |
| 27 | Charles Chapron | 2022 | Charles Chapron (2022) | 220 | 308 | LR | Clinical | 0.7977 | 0.5699 | 175 | 38 | 45 | 50 |
| 28 | Sofiane Bendifallah | 2022 | Sofiane Bendifallah (2022) | 87 | 100 | RF | Clinical | 0.9200 | 0.9200 | 80 | 1 | 7 | 12 |
| 29 | Sofiane Bendifallah | 2022 | Sofiane Bendifallah (2022) | 87 | 100 | LR | Clinical | 0.9500 | 0.8100 | 83 | 2 | 4 | 11 |
| 30 | Sofiane Bendifallah | 2022 | Sofiane Bendifallah (2022) | 87 | 100 | DTA | Clinical | 0.9100 | 0.6600 | 79 | 4 | 8 | 9 |
| 31 | Sofiane Bendifallah | 2022 | Sofiane Bendifallah (2022) | 87 | 100 | XGBoost | Clinical | 0.9300 | 0.9200 | 81 | 1 | 6 | 12 |
| 32 | Sofiane Bendifallah | 2022 | Sofiane Bendifallah (2022) | 87 | 100 | Voter classifier soft | Clinical | 0.9300 | 0.8800 | 81 | 2 | 6 | 11 |
| 33 | Sofiane Bendifallah | 2022 | Sofiane Bendifallah (2022) | 87 | 100 | Voter classifier hard | Clinical | 0.9100 | 0.9200 | 79 | 1 | 8 | 12 |
| 34 | Paula Brady | 2024 | Paula Brady (2024) | 18 | 36 | NN | Genetic | 0.8330 | 0.5830 | 15 | 8 | 3 | 10 |
| 35 | Yu Dai | 2024 | Yu Dai (2024) | 92 | 260 | LR | Clinical | 0.6714 | 0.8794 | 62 | 20 | 30 | 148 |
| 36 | Ping Hu | 2023 | Ping Hu (2023) | 10 | 14 | CNN | Imaging | 1.0000 | 1.0000 | 10 | 0 | 0 | 4 |
| 37 | Ping Hu | 2023 | Ping Hu (2023) | 21 | 31 | CNN | Imaging | 1.0000 | 0.9000 | 19 | 0 | 2 | 10 |
| 38 | V Janša | 2021 | V Janša (2021) | 20 | 26 | SVM | Omics | 0.8100 | 1.0000 | 16 | 0 | 4 | 6 |

**
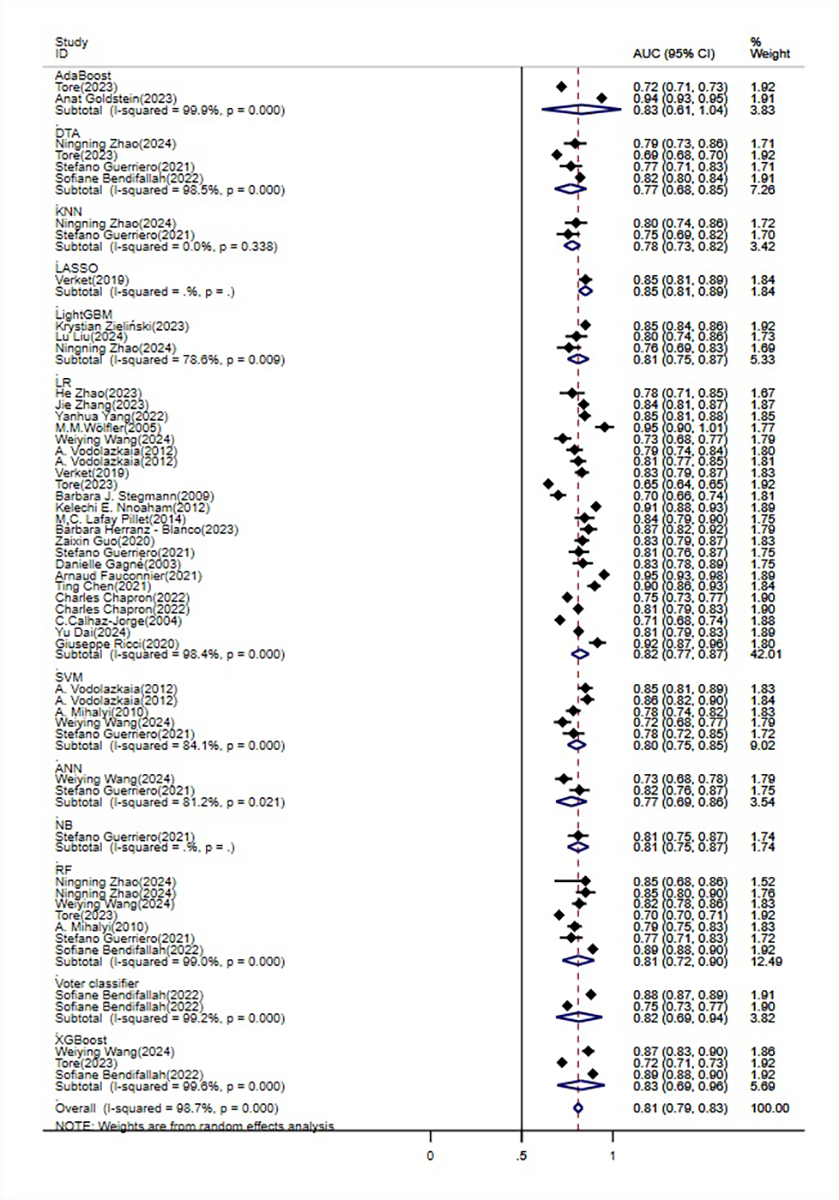
**

**Supplementary** **Figure S1** Forest plot of the meta-analysis for AUC of the clinical feature-based machine learning model in the training set

**
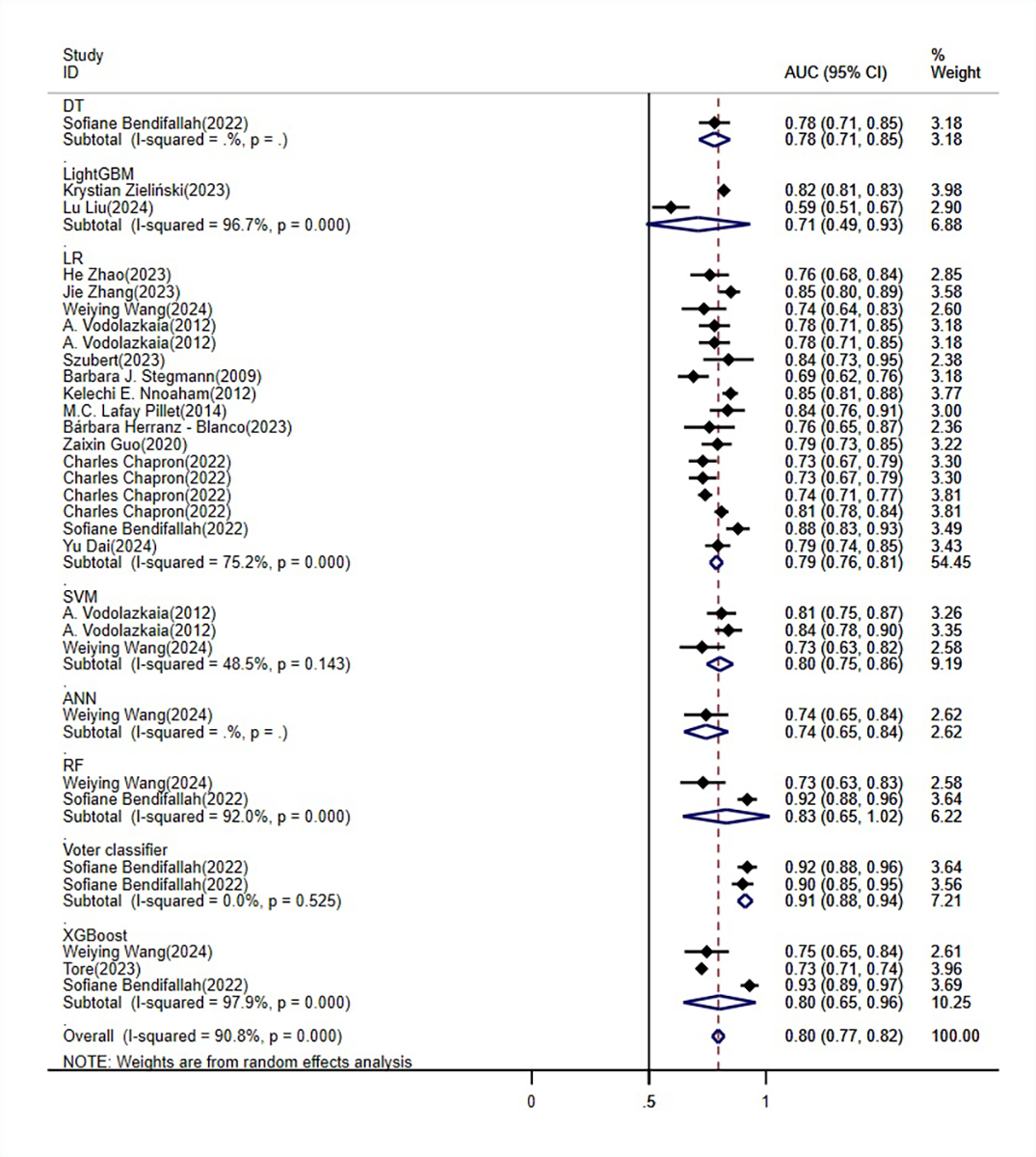
**

**Supplementary** **Figure S2** Forest plot of the meta-analysis for AUC of the clinical feature-based machine learning model in the validation set

**
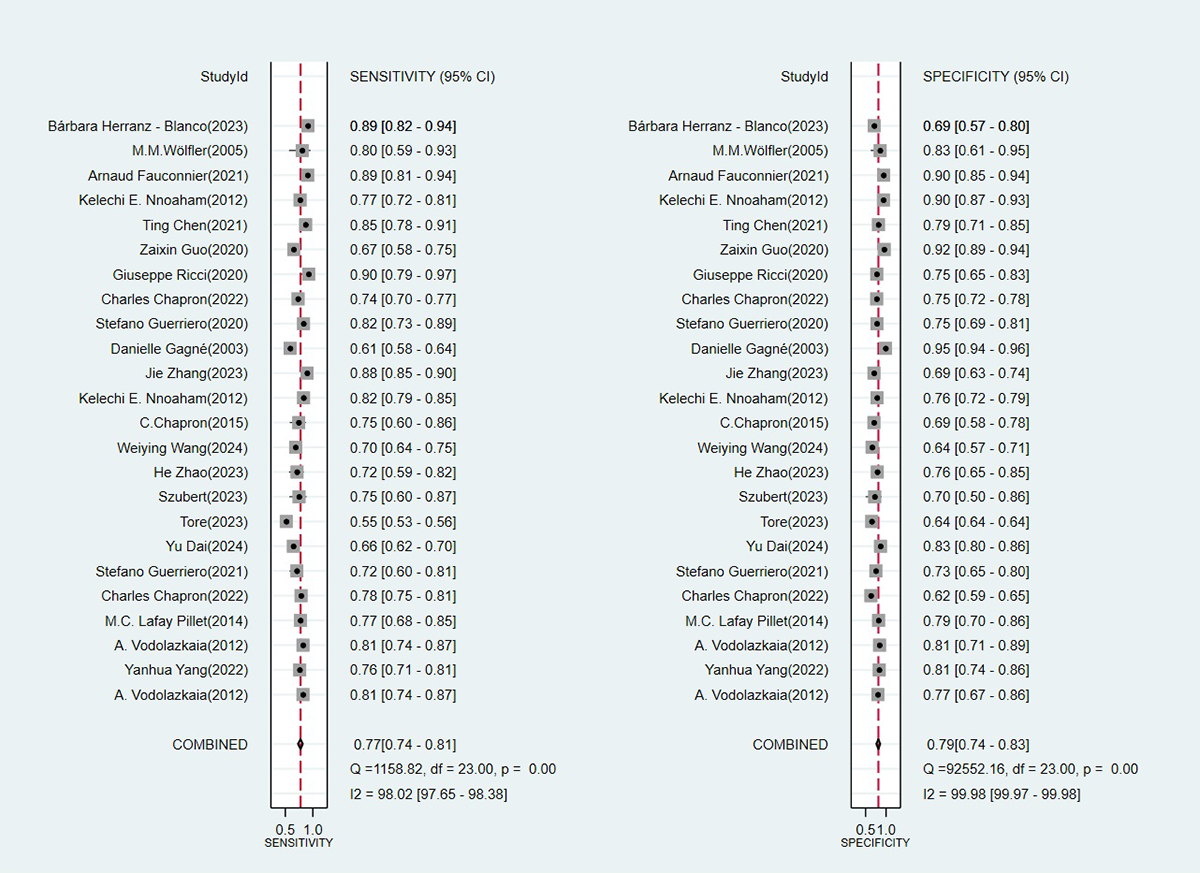
**

**Supplementary** **Figure S3** Forest plot of the meta-analysis for sensitivity and specificity of the clinical feature-based LR model in the training set

**
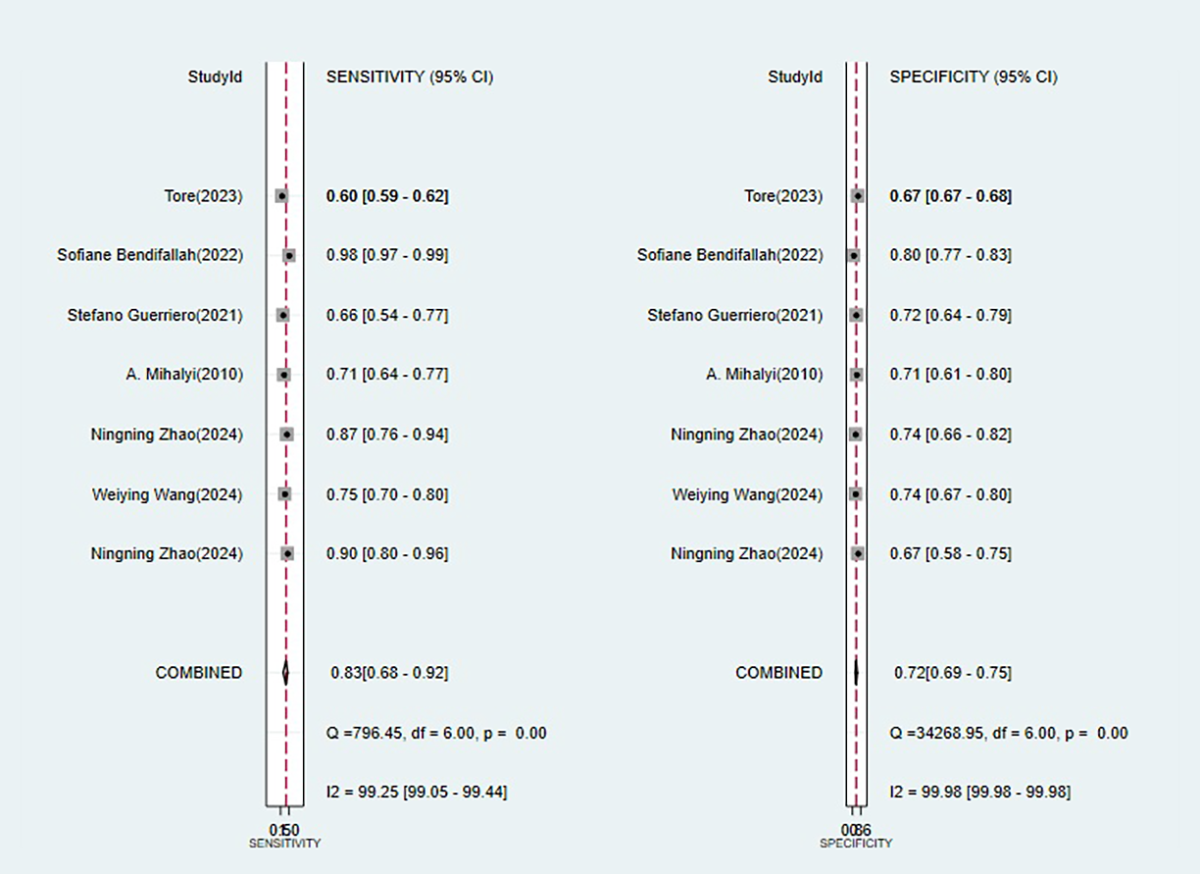
**

**Supplementary** **Figure S4** Forest plot of the meta-analysis for sensitivity and specificity of the clinical feature-based RF model in the training set

**
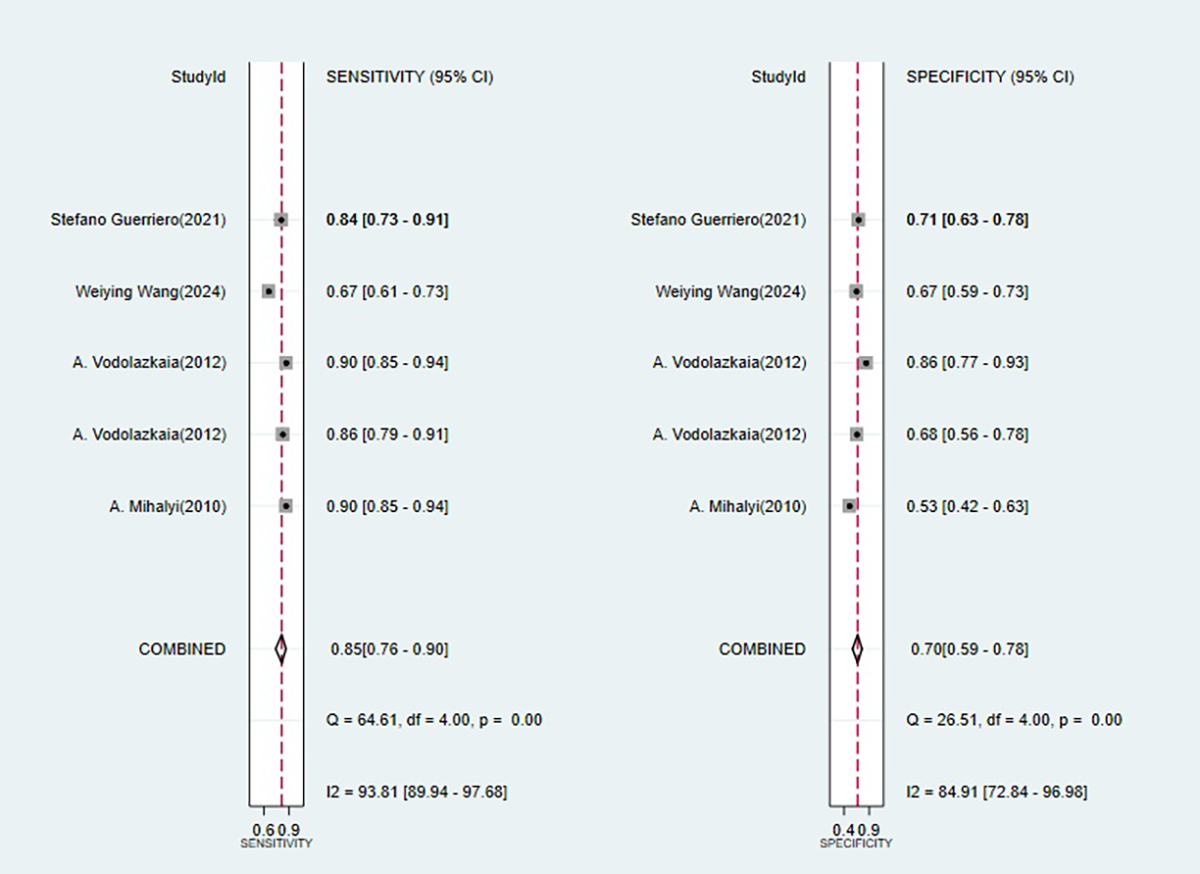
**

**Supplementary** **Figure S5** Forest plot of the meta-analysis for sensitivity and specificity of the clinical feature-based SVM model in the validation set

**
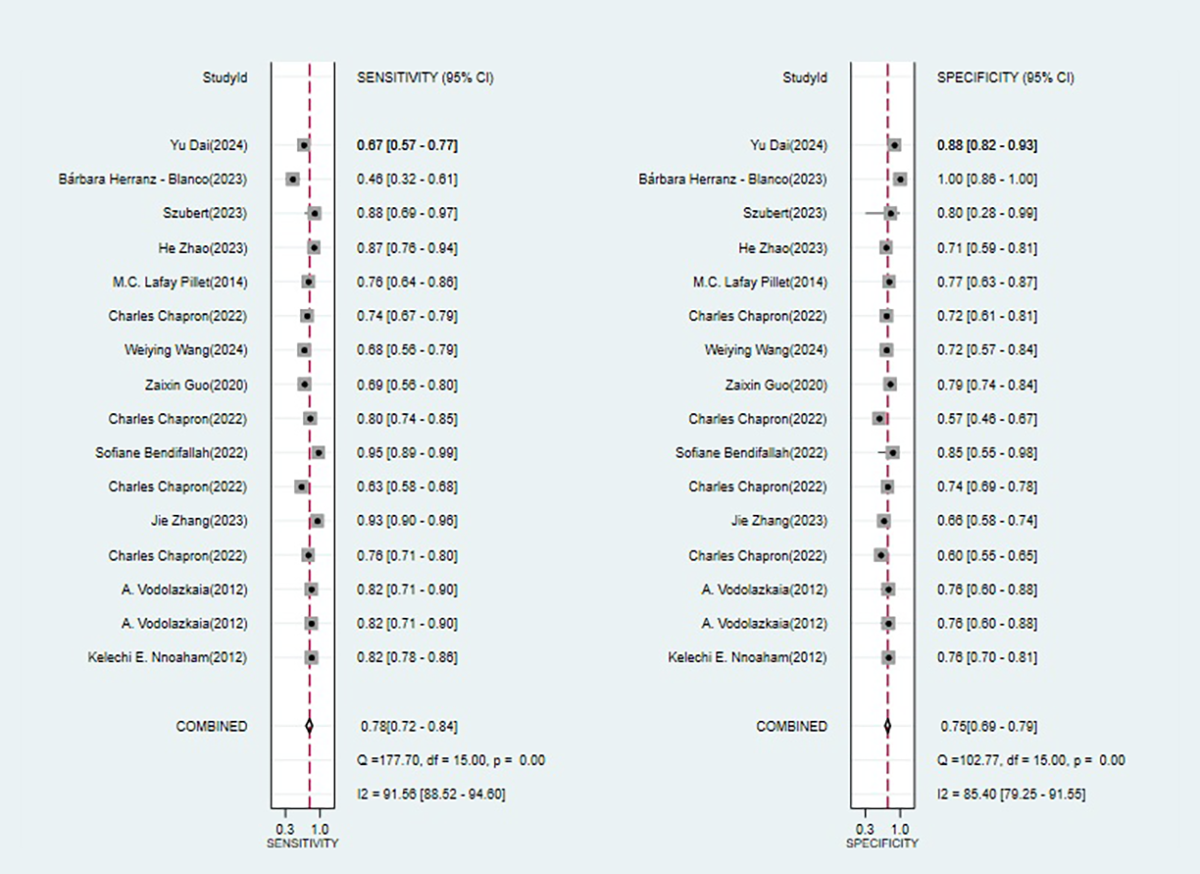
**

**Supplementary** **Figure S6** Forest plot of the meta-analysis for sensitivity and specificity of the clinical feature-based LR model in the validation set
